# Heterocontact-Triggered 1H to 1T′ Phase Transition in CVD-Grown Monolayer MoTe_2_: Implications for Low Contact Resistance Electronic Devices

**DOI:** 10.1021/acsanm.3c01314

**Published:** 2023-07-06

**Authors:** Vladislav O. Khaustov, Domenica Convertino, Janis Köster, Alexei A. Zakharov, Michael J. Mohn, Zewdu M. Gebeyehu, Leonardo Martini, Simona Pace, Giovanni Marini, Matteo Calandra, Ute Kaiser, Stiven Forti, Camilla Coletti

**Affiliations:** † Center for Nanotechnology Innovation @NEST, Istituto Italiano di Tecnologia, Piazza San Silvestro 12, I-56127 Pisa, Italy; ‡ NEST, Scuola Normale Superiore, Piazza San Silvestro 12, I-56127 Pisa, Italy; § Central Facility for Electron Microscopy, Materials Science Electron Microscopy, Ulm University, Albert-Einstein-Allee 11, D-89081 Ulm, Germany; ∥ MAX IV Laboratory, Lund University, P.O. Box 118, Lund, S-22100, Sweden; ⊥ Graphene Laboratories, Istituto Italiano di Tecnologia, Via Morego 30, 16163 Genova, Italy; # Department of Physics, University of Trento, Via Sommarive 14, 38123 Povo, Italy; ○ Institut des Nanosciences de Paris, UMR7588, Sorbonne Université, CNRS, F-75252 Paris, France

**Keywords:** MoTe_2_, monolayer, phase transition, heterocontact, CVD, quantum materials, HRTEM

## Abstract

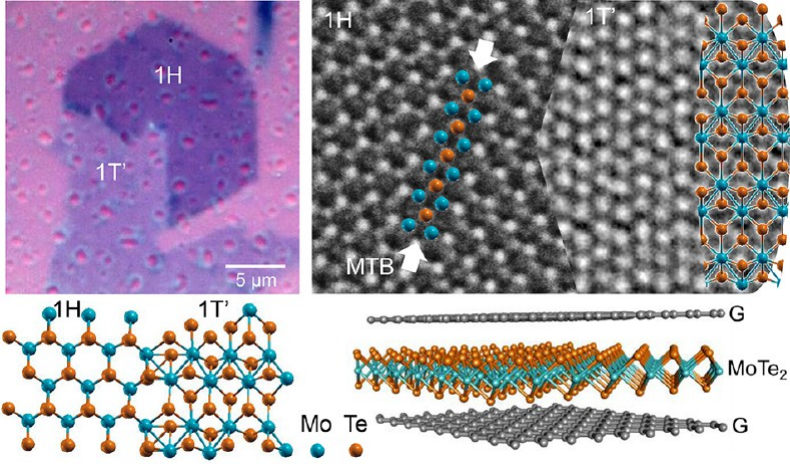

Single-layer molybdenum ditelluride (MoTe_2_) has attracted attention due to the smaller energy difference between the semiconducting (1H) and semimetallic (1T′) phases with respect to other two-dimensional transition metal dichalcogenides (TMDs). Understanding the phenomenon of polymorphism between these structural phases is of great fundamental and practical importance. In this paper, we report a 1H to 1T′ phase transition occurring during the chemical vapor deposition (CVD) synthesis of single-layer MoTe_2_ at 730 °C. The transformation originates at the heterocontact between monoclinic and hexagonal crystals and progresses to either yield a partial or complete 1H to 1T′ phase transition. Microscopic and spectroscopic analyses of the MoTe_2_ crystals reveal the presence of Te vacancies and mirror twin boundaries (MTB) domains in the hexagonal phase. The experimental observations and theoretical simulations indicate that the combination of heterocontact formation and Te vacancies are relevant triggering mechanisms in the observed transformation. By advancing in the understanding and controlling of the direct synthesis of lateral 1T′/1H heterostructures, this work contributes to the development of MoTe_2_-based electronic and optoelectronic devices with low contact resistance.

## Introduction

Molybdenum ditelluride (MoTe_2_) displays structural polymorphism with numerous phases such as 2Ha, 2Hc, 2Hd, 1T′, Td, and 3R existing in the bulk material and the 1H and 1T′ phases stable in the monolayer limit. Because of the lower energy difference (i.e., 30–60 meV/f.u.) between the semiconducting-1H and the semimetallic-1T′ phases^1,2^ with respect to other two-dimensional transition metal dichalcogenides (TMDs), monolayer MoTe_2_ is an extremely attractive candidate for the development of phase change devices^3^ and low-resistance contacts.^4^ Additional interest in these two phases arises as 1H-MoTe_2_ has a direct optical gap of 1.10 eV^5^ and strong spin–orbit coupling,^6^ while 1T′ (Td) exhibits superconductivity in the monolayer limit,^7^ and is predicted to be a 2D topological and large-gap quantum spin Hall (QSH) insulator,^8^ with important implications for the development of spintronic,^9^ valleytronic, near-infrared optoelectronic, and quantum devices. While in the past most of the experiments have been carried out on mechanically exfoliated flakes,^10−14^ nowadays, it has become possible to synthesize 1T′^15^ and 1H as well as several different polymorphs of MoTe_2_, i.e. 2H,^15^ Td,^16^ 3R^17^ and also new forms such as 2D-Mo_5_Te_8_^18^ and 1D-Mo_6_Te_6_ nanowires.^19^ However, while 1H-MoTe_2_ is a well-studied material, 1T′-MoTe_2_ experimental research lags behind due to the extreme air instability of this material, which rapidly degrades upon air exposure, with a lifetime in the minutes range.^20,21^

To date, significant attention has been focused on understanding and achieving controllable 1H/2H to 1T′ structural transformation. Theoretically, different methods have been proposed such as electron/hole injection,^22,3,23,24^ electronic excitation,^25−27^ strain,^28−30^ annealing,^1,22^ chalcogen^31^ or metal^3^ atom substitution, Li^23^ or H^23,32^ doping, and Te vacancies creation.^2,23,27^ Phase transition (PT) has been achieved on few-layers or bulk MoTe_2_ via annealing,^33,10^ ion liquid gating,^34^ electric field in vertical RRAM devices,^11^ Te vacancies creation,^35^ and Li intercalation.^36^ In the monolayer limit, investigations on 1H/1T′ phase transition are highly complicated by the air instability of monolayer 1T′, although promising initial results have been reported by adopting annealing,^10^ ionic liquid gating,^34,37^ THz laser irradiation^38^ and Te vacancies creation.^39^ While developing approaches for triggering 1H–1T′ phase transition with external stimuli remains of undoubted interest for the realization of phase change devices, progressing in understanding and controlling the direct synthesis of lateral 1T′/1H heterostructures is the base for the development of low resistance contacts for electronics and optoelectronics and for devising novel device architectures. Nowadays, growth of 1H/1T′ lateral heterostructures has been demonstrated only in UHV conditions via Mo and Te evaporation on conductive HOPG^18^ or graphene substrates.^40^ The 2H–1T′ phase transition critical temperature, a relevant aspect in the controlled synthesis of the two phases, is still an open question. For bulk MoTe_2_ crystals, the pioneering work of Velinga et al.^33^ reported a critical temperature of 850 °C, while Keum et al.^41^ demonstrated critical temperatures between 500 and 700 °C, with the latter result being debated in the literature.^42^ Remarkably, measuring the transition temperature is even more challenging for monolayer MoTe_2_ films due to the low material stability, as Te vacancies and 1D-Mo_6_Te_6_ chains formation were observed already at 200 °C.^12^ Recently, the transition temperature of monolayer MoTe_2_ encapsulated with hexagonal boron nitride (hBN) has been measured to be 1075 °C.^10^

In this paper, we demonstrate that monolayer 1H/1T′ MoTe_2_ lateral heterostructures can be obtained via chemical vapor deposition (CVD) directly on SiO_2_/Si, thanks to a heterocontact-triggered structural phase transition taking place at 730 °C during the growth process. The resulting 1H/1T′ crystals are stabilized with a scalable encapsulation approach recently reported by our group^20^ and their structural and chemical properties are investigated by Raman spectroscopy, high-resolution transmission electron microscopy (HRTEM) and X-ray photoemission spectroscopy (XPS). Experimental data indicate that the phase transition is triggered independently from the contact angle and reveal the presence of Te vacancies and mirror twin boundary (MTB) domains in the hexagonal phase. Ab initio calculations support the observed phenomenology by indicating that defects such as Te-vacancies favor phase transition, while the presence of MTB and excess Mo are possibly responsible for its termination. The final 1H/1T′ contact fronts can have a lateral size in the range of tens of micrometers, which is relevant for device fabrication such as field effect transistors (FETs) and photodetectors. The results presented are of use to devise and engineer tailored 1H/1T′ lateral heterostructures for the development of novel phase-change, spintronic, and quantum devices with reduced contact resistance compared to fabricated metallic contacts.

## Experimental Section

### Growth of Monolayer MoTe_2_

The MoTe_2_ samples were grown via liquid precursor CVD, as we previously reported in Pace et al.^20^ In this method, the molybdenum precursor is obtained from an aqueous solution and directly spun on the SiO_2_ substrate. First, three mother solutions were prepared, namely, solutions A, B, and C. Solution A was obtained by dissolving 0.11 g of ammonium heptamolybdate (AHM, Sigma-Aldrich), weighted by analytical balance ABJ 80-4NM, in 40 mL of DI water. Solution B was obtained by dissolving 0.1 g of NaOH (Sigma-Aldrich) in 40 mL of DI water. Solution C consists of OptiPrep (Sigma-Aldrich) used as purchased. The growth solution was obtained by mixing the mother solutions with volume ratio A:B:C = 0.8:0.5:0.3. The growth solution was then spin-coated on a clean SiO_2_/Si substrate at 300 rpm for 10 s, plus 20 s at 3000 rpm. Before spin-coating, all SiO_2_ substrates were cleaned via sonicated cleaning in acetone and isopropanol for 5 min and oxygen plasma (power = 100 W, process pressure = 80 mTorr) for 5 min to increase the hydrophilicity of the SiO_2_ surface. The spin-coated substrate and metallic tellurium were then loaded in a Lenton hot-wall horizontal CVD reactor (see Figure S1 for furnace schematic). The growth was carried out at near-atmospheric pressure for 2 to 15 min, under a constant flow of Ar/H_2_ (H_2_ 3%) gas at 100 sccm. Growth temperature, molybdenum concentration, and cooling process were optimized to increase the density of spontaneously contacting hexagonal and elongated MoTe_2_ flakes as well as material thickness uniformity. Best results were obtained at a growth temperature of 730 °C, with increased molybdenum precursor concentration, and by opening the CVD reactor at 700 °C. After the growth, the reactor was allowed to cool rapidly under constant flux of Ar by opening the lid at 700 °C. We note that in our process the 1H to 1T′ transformation is of a rapid “explosive” nature; i.e., the percentage of the transformed area of hexagonal flakes undergoing phase transition is independent of the growth time, which can be reduced down to 2 min (see Figure S2). In our experiments, for consistency, we adopt a growth time of 15 min. We report that growth time reduction below 2 min leads to the synthesis of incomplete crystals.

### Semidry Encapsulation

Semidry top-encapsulation of as-grown MoTe_2_ was obtained via the delamination of CVD hBN or graphene films grown on copper foil. Nominally, single-layer hBN (15 × 15 cm^2^) from Graphene Supermarket was used for encapsulation. Graphene samples were grown in-house using a 4″ Aixtron BM Pro CVD reactor.^43^ The hBN (graphene) was cut into squares with dimensions larger than the target SiO_2_ sample. Then, it was covered with a double-layer polymeric membrane of PMMA AR-P 679.02 (Allresist) and 15% PPC in anisole (Sigma-Aldrich) by spin coating for 60 s at 2000 rpm and baked at 90 °C for 2 min. A PDMS frame with few millimeters of thickness was placed on the top of the sample as a supporting frame. The sample was then floated in a NaOH electrolyte solution (1 M), where an electrochemical reaction takes place: here the hBN (graphene) on copper foil acts as anode and a platinum foil is used as cathode. A constant 2.42 V voltage is applied to the Pt electrode until the PPC/PMMA/hBN(graphene) is completely delaminated from copper foil. Then the PPC/PMMA/hBN(graphene) was allowed to float in DI water for 3 min to remove the NaOH residuals. The detached PPC/PMMA/Graphene membrane was then laminated on top of the as-grown sample and heated at 90 °C. Finally, a double cleaning in acetone and isopropanol was used to remove the supporting polymer.

### MoTe_2_ Transfer

Graphene or hBN top-encapsulated MoTe_2_ was transferred using HF (Sigma-Aldrich) as SiO_2_ etchant, as previously reported in.^20^ In particular, the sample (i.e., encapsulated MoTe_2_ on SiO_2_/Si) was covered with a double-layer of 679.02 PMMA (Allresist), which was sequentially spun (60 s, 2000 rpm) and baked (90 °C, 2 min). Then, the sample was left floating in concentrated hydrofluoric acid (HF 48%, Sigma-Aldrich) for few seconds until the complete detachment of the membrane. After detachment, the floating membrane was fished and rinsed in DI water for a few seconds to minimize the exposure of MoTe_2_ to water. Finally, the membrane was rapidly fished with the target substrate (silicon for XPEEM measurements and 100 nm SiO_2_/Si for TEM sample preparation) and backed at 90 °C for a few minutes. The polymeric membrane was then removed via a standard cleaning in acetone. Indeed, often the formation of a parasitic Te-like phase was observed in the monolayer 1T′ regions upon the transfer process,^44,45^ hence in this work we limited our PEEM/XPS analysis to the 1H and 2H regions.

Graphene top-encapsulated MoTe_2_ transferred on a 100 nm SiO_2_ substrate was used for TEM sample preparation. Optical microscopy was used to identify suitable MoTe_2_ monolayers. Such flakes were then transferred to a Quantifoil R 1.2/1.3 TEM grid with the help of a drop of isopropyl alcohol (IPA) to bring the grid into contact with the flake. Evaporation of the IPA attached the grid to the flake. With potassium hydroxide (KOH), the underlying SiO_2_ substrate was etched away, releasing the grid with the MoTe_2_ flake. Afterward, the residues of the preparation are removed with double-distilled water.

### Characterization Techniques

The optical images of the samples were obtained with a ZEISS Axioscope 7. Amplitude modulated Kelvin probe force microscopy (AM-KPFM) was performed with a Bruker Dimension Icon microscope equipped with an FMV-PT probe without applying bias to the tip. Raman characterization was carried out with a Renishaw InVia spectrometer. The laser wavelength used was 532 nm with a fwhm of the Gaussian beam ∼1600 or 800 nm for 50× or 100× lenses, respectively, in long exposure time mode (60/180 s) and low power (1.16/0.58 mW) and in short exposure time (1–2 s) and high power (5.8 mW) mode. Polarized Raman measurements were implemented in *z*(*x*,−)*z̅* configuration using a λ/2 waveplate for the incoming light.

X-ray photoemission electron microscopy (XPEEM) measurements were performed by using the ELMITEC-LEEM III instrument at the MAXPEEM beamline of the MAX-Lab synchrotron radiation facility in Lund, Sweden. The measured photocurrent is 20 mkA at 100 eV and 70 mkA for 350 eV. The work function of the analyzer is 4.8 eV. High-resolution (HR)TEM images were acquired with the *C*_c_/*C*_s_-corrected “Sub-Ångström Low-Voltage Electron microscope” (SALVE)^46^ at an acceleration voltage of 80 kV. Measured values for the chromatic aberration *C*_c_ and spherical aberration *C*_s_ were in the range of −10 μm to −20 μm. The vacuum pressure in the column of the TEM was on the order of 1 × 10^–5^ Pa.

Scanning transmission electron microscopy (STEM) and energy-dispersive X-ray spectroscopy (EDX) were carried out to determine the local elemental composition. For the STEM-EDX measurements, a Thermo Fisher Talos 200X (S)TEM was operated at 80 kV. The system is equipped with a SuperX EDX detector for spectroscopy and elemental mapping.

### Computational Methods

Density Functional Theory (DFT) calculations were performed in the open-source Quantum ESPRESSO (QE) package.^47^ The exchange-correlation functional was described by the modified Perdew–Burke–Ernzerhof (PBEsol) version of the generalized gradient approximation (GGA) with scalar-relativistic Optimized Norm-Conserving Vanderbilt and Ultrasoft pseudopotentials used for Mo and Te atoms, respectively.^48−50^ The kinetic energy cutoff for wave functions was set to 45 Ry. We used Marzari–Vanderbilt–Devita–Payne cold smearing approach for electronic occupations with 0.1 eV.^51^ A 12 × 7 × 1 Monkhorst–Pack wave-vector grid was used for both rectangular 1H and 1T′ cells with an 18.729 Å vacuum thickness for both 1H and 1T′ phases. Vc-relaxed lattice parameters were calculated to be *a* = 3.505 Å, *b* = 6.07 Å for 1H and *a* = 3.398 Å, *b* = 6.307 Å for 1T′ pristine cells containing 6 atoms (2 Mo + 4 Te). We used 3 × 2 × 1, 7 × 1 × 1, and 1 × 7 × 1 supercells with 4 × 4 × 1, 2 × 7 × 1, and 12 × 1 × 1 *k*-space grids, respectively, to simulate Mo diffusion and heterocontact-induced phase transitions in monolayer MoTe_2_. For the orthogonal b_1T′_/2a_1H_ heterocontact case, we used a combination of 3 × 1 × 1 and 2 × 2.5 × 1 supercells of 1T′ and 1H phases, respectively, with 2 × 7 × 1 *k*-space grid. To minimize the periodic effect of the replicas on the studied heterointerface, the atoms of the first and last “subcells” of the supercells were kept fixed in the positions obtained from the pristine 1H and 1T′ relaxed supercells at equilibrium cell size. In the case of the ZZ supercell, 1 Te atom from the supercell edge was removed because of the small Te–Te distance at the supercell boundary. Te-deficient heterocontact cells were relaxed using “epitaxial_bc/ac” method to avoid additional out of plane deflections. The climbing image nudged elastic band method (CI-NEB) was performed to calculate kinetic barriers between the states. Phase diagrams calculations were performed using linear elasticity theory.^52^ Elastic energy difference was calculated using elastic constants *C*_*ij*_ calculated by applying deformations reported in the work.^53^

## Results and Discussion

**Figure 1:**
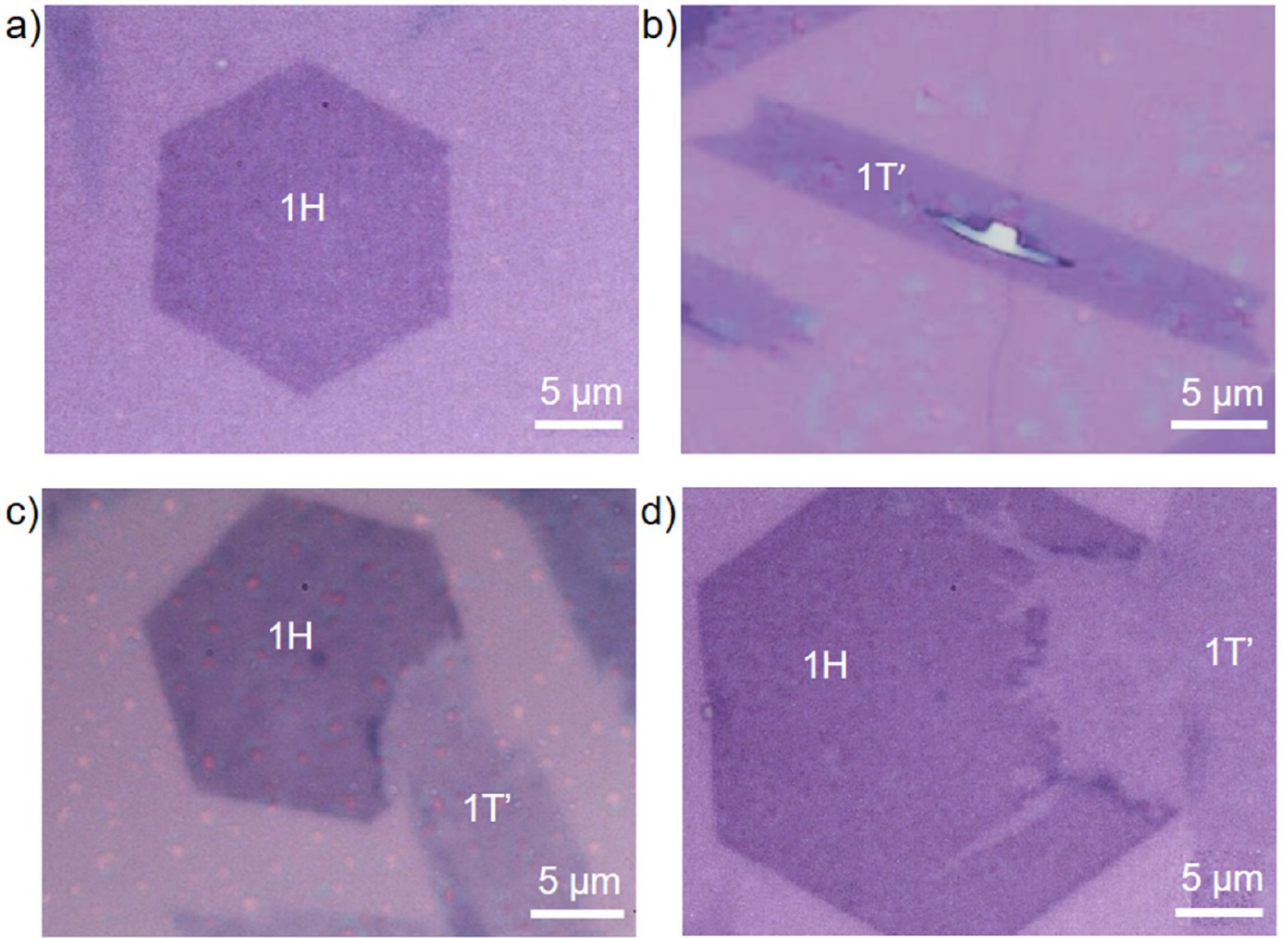
Optical images of pristine hexagonal 1H single crystal (a), pristine monoclinic 1T′ single crystal (b), heterocontact between 1H and 1T′ single crystals with uniform (c), and nonuniform 1H to 1T′ transformation (d).

**Figure 2:**
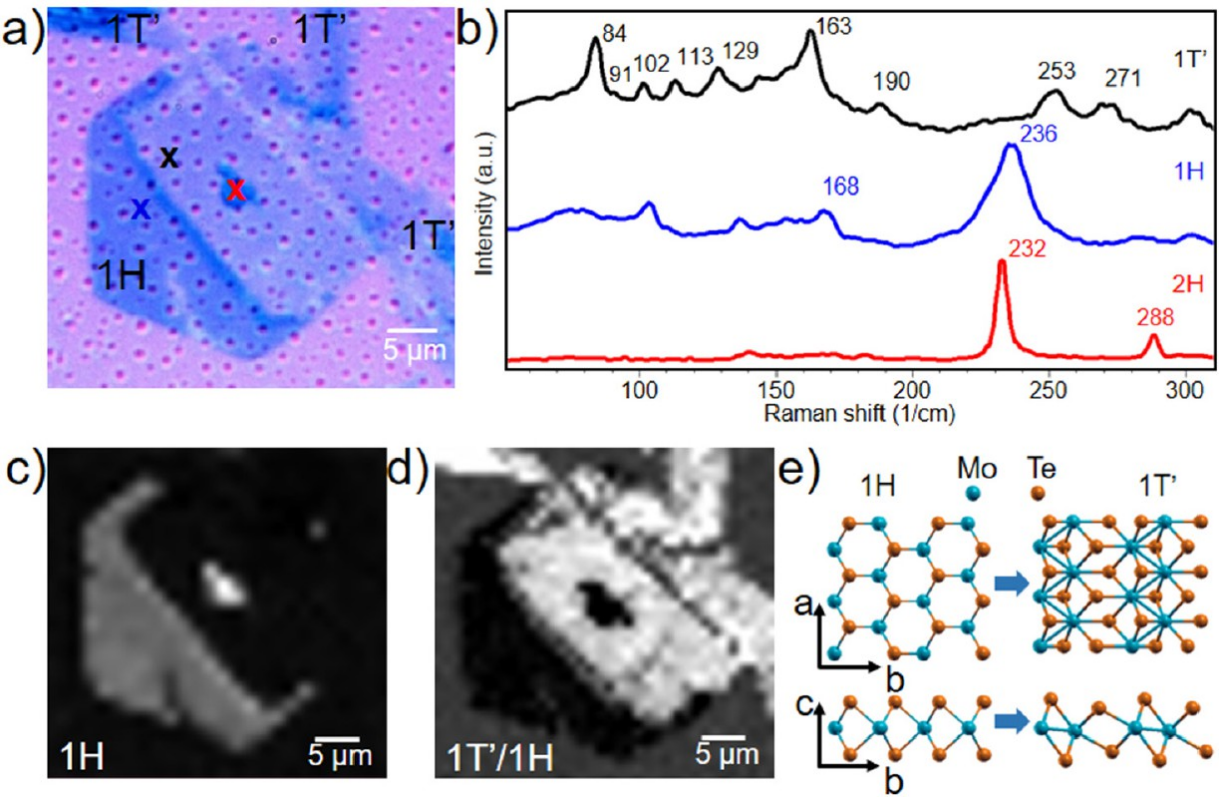
(a) Optical image of a flake presenting a heterocontact-triggered phase transition. (b) Raman spectra recorded on the blue, red, and black crosses in part a. (c) Map of the intensity of the 1H 236 cm^–1^ E_2g_ Raman peak. (d) Map of the intensity ratio of the Raman 84 cm^–1^ A_g_^1^ (1T′) and 236 cm^–1^ E^1^_2g_ (1H) peaks. (e) Top and side views of 1H and 1T′-MoTe_2_ ball-and-stick models; blue (orange) colors indicate the Mo (Te) atoms. The well-visible crystal break in Figure 2a,d running from side to side of the hexagonal flake originated during hBN encapsulation, and it does not influence the crystallographic orientation of the 1T′ transformed hexagonal crystal.

In Figure 1a,b, we show the optical micrograph of typical crystals of MoTe_2_ grown on SiO_2_/Si via liquid precursor CVD. Crystals of different shape are visible within the same sample: hexagonal (a) and elongated (b) ones, which are normally attributed to the 1H and 1T′ phase, respectively. In fact, 1H-MoTe_2_ is stable in a hexagonal phase (space group *P*6*m*2^22^) and hence grows with hexagonal symmetry, while 1T′-MoTe_2_ is stable in a monoclinic structure (space group *P*2_1_/*M*^22^) and grows in an elongated shape. We optimized the growth parameters to maximize the density of MoTe_2_ flakes in each sample, which in turn led to the observation of a significant number of heterocontacts between hexagonal and elongated flakes (like those shown in Figures 1c,d and 2a). The MoTe_2_ samples were encapsulated immediately after growth with monolayer CVD hBN (or graphene) to increase the lifetime of 1T′-MoTe_2_ and allow further characterization, similar to what reported in.^20^

In Figure 2a, one can observe three elongated flakes contacting a hexagonal crystal, which interestingly presents different optical contrasts. A precise assignment of the structural phase of the material, carried out by Raman spectroscopy indicates that only part of the hexagonal flake displays a 1H phase, with the remnants being 1T′ (see Figure 2b–d). Indeed, Figure 2b reports the typical Raman spectra measured in different regions (marked with crosses) of panel a. The blue and black spectra were recorded on the regions with dark and light contrast, respectively, while the red spectrum was recorded at the center of the hexagonal crystal. The blue spectrum shows typical features of a monolayer 1H phase, such as the in-plane E^1^_2g_ mode at 236 cm^–1^ and the out-of-plane A_1g_ mode at 168 cm^–1^,^54^ while few-layer peaks E_1g_ at 116 cm^–1^ and B^1^_2g_ at 288 cm^–1^ are absent.^13,55^ The portion of the hexagon with darker contrast in the left side of panel a is hence assigned to 1H-MoTe_2_. The triangular feature with darker contrast placed at the center of the hexagon presents a Raman spectrum that is indicative of few-layer 2H-MoTe_2_, as typically observed in seeding areas. The portion of the hexagon with lighter contrast presents a Raman spectrum with six A_g_ peaks located at 84, 113, 129, 163, 253, and 271 cm^–1^ and three B_g_ peaks positioned at 91, 102, and 190 cm^–1^, which are indicative of monolayer 1T′.^56^ Indeed, the spatial distribution of the two different phases is very visible with spatially resolved Raman mapping. Panels c and d report Raman maps of the intensity of the 236 cm^–1^ E^1^_2g_ peak and of the intensity ratio of the 84 cm^–1^ A_g_^1^ and 236 cm^–1^ E^1^_2g_ peaks for a straightforward visualization of the 1H and 1T′ phases, respectively (ball-and-stick models of the two phases are sketched in panel e). The presence of a 1T′ region within the hexagonal crystal is indicative of a phase transition taking place during the growth process itself, which is performed at a maximum temperature of 730 °C. As also supported by the data presented in the rest of the paper, the transformation is triggered by the presence of a direct contact between a 1H and a 1T′ crystal. Indeed, we confirm this phenomenology in many samples (>100), where hexagonally shaped flakes contacted by elongated ones present partial or total transformation to 1T′ (see Figure S3). The observed PT mechanism is enticing as it leads: (i) to the generation of large lateral 1H/1T′ heterostructures (i.e., tens of micrometers, significantly larger than those typically found between randomly touching flakes); (ii) to full transformation of large 1T′-MoTe_2_ hexagonal crystals.

**Figure 3:**
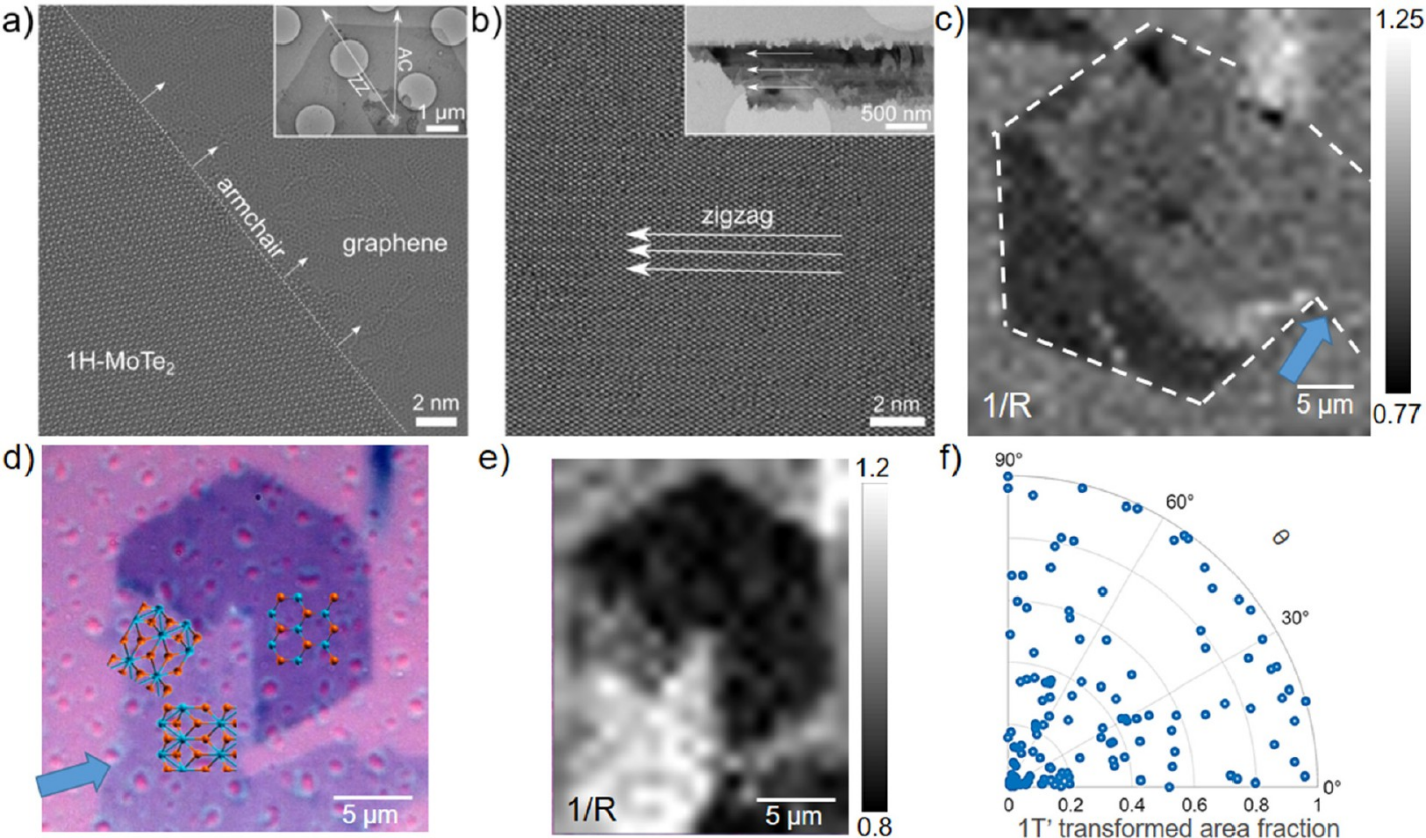
(a) 80 kV *C*_c_/*C*_s_-corrected HRTEM image of an untransformed 1H flake edge with identified AC direction. In the inset: TEM overview image of the 1H hexagon. (b) 80 kV *C*_c_/*C*_s_-corrected HRTEM image of a 1T′ flake with identified ZZ direction. Inset: TEM overview image of the monoclinic 1T′ crystal. (c) 1/*R* Raman mapping of the flake studied in Figure 1. (d) Optical image of a partially transformed flake in the case of collinear contact with ball-and-stick models of the identified crystal orientations. (e) 1/*R* Raman mapping of the flake in panel d. f) Collected statistical data of the percentage of hexagonal flakes transformed into 1T′phase with respect to the heterocontact angle (θ), i.e., the angle between 1H and 1T′ ZZ directions.

To better understand the crystal orientation of the contacting and transformed 1T′ flakes, we adopted a combination of high-resolution transmission electron microscopy (HR-TEM) and polarized Raman spectroscopy measurements. Indeed, polarized Raman spectroscopy has been intensively used in the study of bulk,^57−59^ few-layer^59,60^ and monolayer^56^ MoTe_2_ optical and crystallographic properties, as the A_g_^5^ (253 cm^–1^) and A_g_^6^ (271 cm^–1^) modes allow defining the 1T′ crystal direction, if excited with linearly polarized light.^61^ The maximum value of the intensity ratio I(A_g_^5^)/I(A_g_^6^)—which we refer to as *R*-value in the following text—corresponds to the [0 ± π] angle between the incident light polarization and the crystal’s zigzag (ZZ) direction.^56,61^ HR-TEM allowed us to identify the edges of our untransformed hexagonal 1H crystals to be armchair (AC) terminated, while 1T′ single crystals were found to elongate along the Z*Z*-direction (see Figure 3a,b). Via polarized Raman, we measured a maximum *R*-value in 1T′ flakes in horizontal configuration (i.e., long side/ZZ direction parallel to the light polarization), with the minimum measured for the orthogonal alignment configuration (see Figure S4). For the flakes displayed in Figure 2a, the spatial distribution of the R-value allowed us to determine that the dominating crystallographic direction of the transformed hexagonal crystal corresponds to that of the lower contacting 1T′ flake (see Figure 3c and Figure S5). This finding suggests that the 1H–1T′ phase transition was originally triggered by the flake indicated in Figure 3c by the light blue arrow.

We verified with a large number of flakes and found that in all instances the transformed 1T′ crystals have a direction that is either collinear or 60° rotated with respect to that of the original 1T′ contacting flake. Figure 3 report the case of transformation with collinear orientations of initial 1H and 1T′ crystals (i.e., Z*Z*-directions originally parallel in both crystals), where we see the formation of a 60° rotated 1T′ domain and the incompleteness of the transition process. We argue that such polycrystallinity observed in some of the flakes (see also Figures S6 and S7) is a consequence of the 1H 3-fold rotational symmetry that might lead to three different 1T′ crystal orientations rotated by 60°.^62^

Also, we note that not all contacts between elongated 1T′ and hexagonal 1H crystals result in structural phase transitions, while in some instances a limited local recrystallization near the contact points is observed. To better understand the cause of the heterocontact-induced phase transformation phenomenon, we have investigated whether there is any preferential contact angle between the 1T′ flake and the hexagonal crystal that leads to the transition and whether this can be related to the percentage of the transformed flake. By inspecting 99 heterocontacts, we can conclude that there is no such indication: neither the occurrence of the phase transition nor the percentage of the transformed 1H flake is obviously related to the initial contact angle (see Figure 3f).

**Figure 4:**
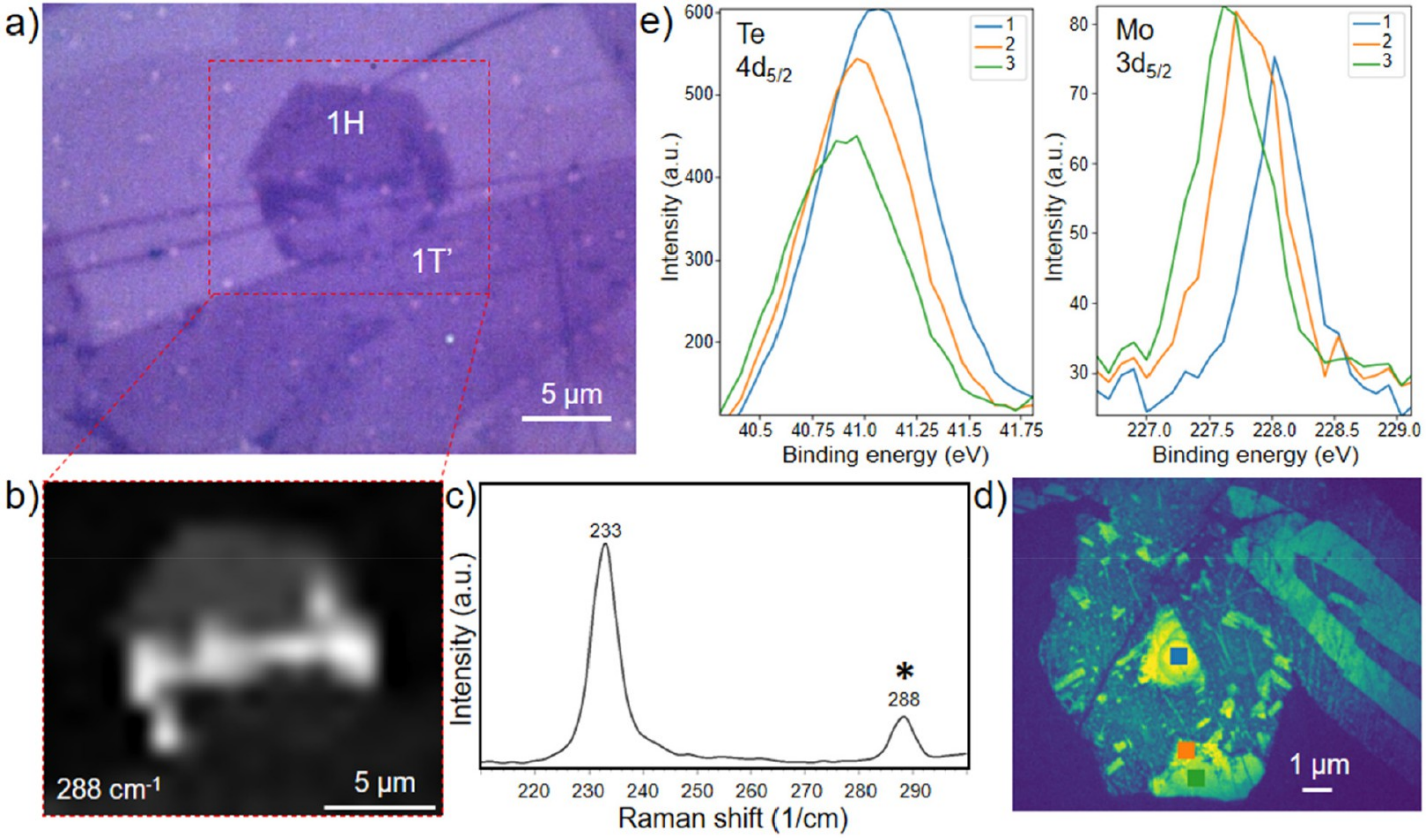
(a) Optical image and (b) B^1^_2g_ mode Raman mapping of a partially converted MoTe_2_ crystal. (c) Raman single spectrum obtained from the heterocontact region of the flake in parts a and b confirming the presence of few-layers 2H. (d) Te 4d μXPEEM image and (e) relative Te 4d_5/2_ and Mo 3d_5/2_ intensity profiles.

The optical micrographs in Figures 2a and 3d present darker contrast at the 1H–1T′ interface, compatible with few-layer MoTe_2_. This is also visible in Figure 4a, where we show an optical image of a multiphase MoTe_2_ flake, where Raman analysis of the optically darker region reveals few-layer 2H features. Indeed, the B^1^_2g_ mode at 288 cm^–1^ is observed (panel b) while the E^1^_2g_ mode shifts from 236 to 233 cm^–1^ (panel c).^13^ Such few-layers regions observed at the 1H–1T′ interface suggest the presence of excess molybdenum in those areas during the CVD process, which transforms into few-layer MoTe_2_ as tellurium is fluxed in the tube. Similar evidence of excess Mo diffusion during the CVD process is found in several multiphase MoTe_2_ hexagonal flakes presenting elongated lobes at the edge of 1T′ transformed portions (see Figure S8). While the stoichiometry during the growth process cannot be verified but only indirectly inferred, more information on the chemical composition of the grown flakes can be extracted via X-ray photoemission spectroscopy (XPS) measurements, which were performed at the X-ray photoemission electron microscopy (XPEEM) beamline of the MAX-Lab synchrotron. To this end, graphene-encapsulated MoTe_2_ samples transferred on silicon were analyzed. In Figure 4e, we show the Te 4d and Mo 3d core levels measured on the areas indicated by colored squares in the Te 4d XPEEM map (panel d). We analytically extracted the Mo:Te ratios for three different regions: 2H seed (blue square), 1T′–1H heterocontact boundary (orange square), and 1H region (green square). The ideal Mo:Te stoichiometry of the pristine MoTe_2_ is equal to 0.5. We obtain a value of 0.43 ± 0.04 for the 2H seed area, 0.51 ± 0.04 for heterocontact area, and 0.57 ± 0.05 for the monolayer 1H region (see Supporting Information for more information). These values and the Te 4d_5/2_ intensity variation suggest the presence of Te vacancies in the untransformed 1H area.

**Figure 5:**
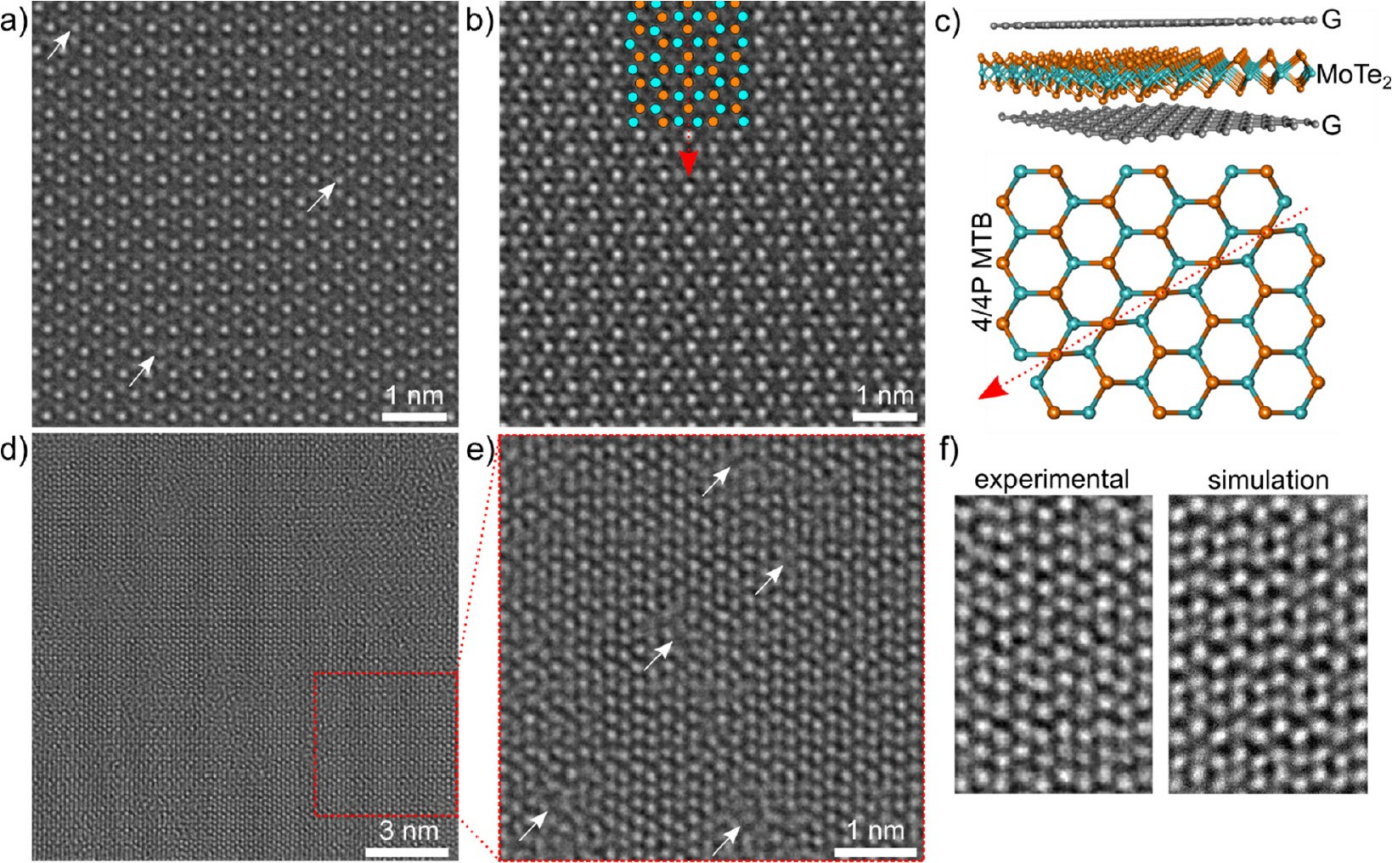
80 kV *C*_c_/*C*_s_-corrected HRTEM analysis of monolayer 1H and 1T′ MoTe_2_. Images of (a) Te single vacancies and (b) 4/4P MTB in the nontransformed 1H area. (c) Schematic of the graphene-encapsulated MoTe_2_ and ball-and-stick model of the 4|4P MTB in part b. Blue (orange) balls correspond to the Mo (Te) atoms. (d) Atomically resolved image of monolayer 1T′ crystal showing the presence of defects. (e) Magnified region from part d. Defective regions are marked with white arrows. (f) Experimental and simulated HRTEM images of graphene-encapsulated monolayer 1T′ phase (see Figures S11 and S12 for corresponding selected area electron diffraction patterns and additional image simulations, respectively).

High-resolution transmission electron microscopy (HRTEM) was performed to validate the crystallinity of the flakes on the atomic scale and confirm the presence and nature of defects. To minimize beam damage in the monolayers, MoTe_2_ was encapsulated with graphene. Monolayer 1T′ showed a rather defective crystalline structure (Figure 5d,e) and the heterocontact region could not be imaged (Figure S10d–f), due to damage arising during transfer to the grid because of the high sensitivity of the 1T′ phase to oxygen.^63,64^ Conversely, in the 1H region, two types of defects were identified: (i) single Te vacancies (see Figure 5a); (ii) inversion domains, mainly 4|4P Mirror Twin Boundary (MTB) domains (Figures 5b,c and S10). These MTBs have already been observed in experiments with Mo excess forming “triangular” and “wagon-wheel” shaped domains,^65^ and in the newly reported hexagonal 1H–Mo_5_Te_8_ phase,^18^ ultimately confirming the Te deficiency already highlighted by XPEEM measurements.

Our experimental data indicate that heterocontact-triggered polymorphism takes place: (i) independent from the 1T′/1H contact angle; (ii) in the presence of Te vacancies and MTB within the 1H original crystal; (iii) during CVD growth and while Mo is diffusing.^66^ In the following text, we adopt linear elasticity theory^52^ and ab initio calculations to model and further understand the observed phenomenology.

Before entering into this discussion, we consider the effect of the strain, originating from the 1H–1T′ lattice constant mismatch, on the polymorphism reported in this work. Our density-functional theory (DFT) simulations indicate 1.19% compression along ZZ and 1.56% stretching along AC as typical strain values for a 1H/1T′ HC in the collinear case. The calculated strain values correspond to small variations in the energy difference between the two phases of the order of few meV/f.u. compared to the calculated 60 meV (30 meV/f.u.) ground state energy difference between relaxed 1H and 1T′ cells (see Figure S13). This indicates that strain is not likely to play a determinant role in the transformation. This is also confirmed by experimental observations: if the strain was the dominant mechanism triggering the PT, we would expect to measure a maximum of the 1H–1T′ transition probability density as a function of the angle, in correspondence to 60° periodicity, where the calculated energy difference between the two phases is minimum (see Supporting Information for discussion and Figure S13). Considering additional 0.66% biaxial strain from the thermal expansion,^67^ this angle corresponds to the collinear ZZ directions of 1T′ and parent 1H crystals, in agreement with previous theoretical predictions for this system.^29^ In contrast, our experimental data do not show a univocal angle dependence; rather, a uniform probability density for the occurrence of the phase transition is observed (see Figure 3f). Thus, we conclude that the strain effect is not determinant in triggering the phase transition.

**Figure 6:**
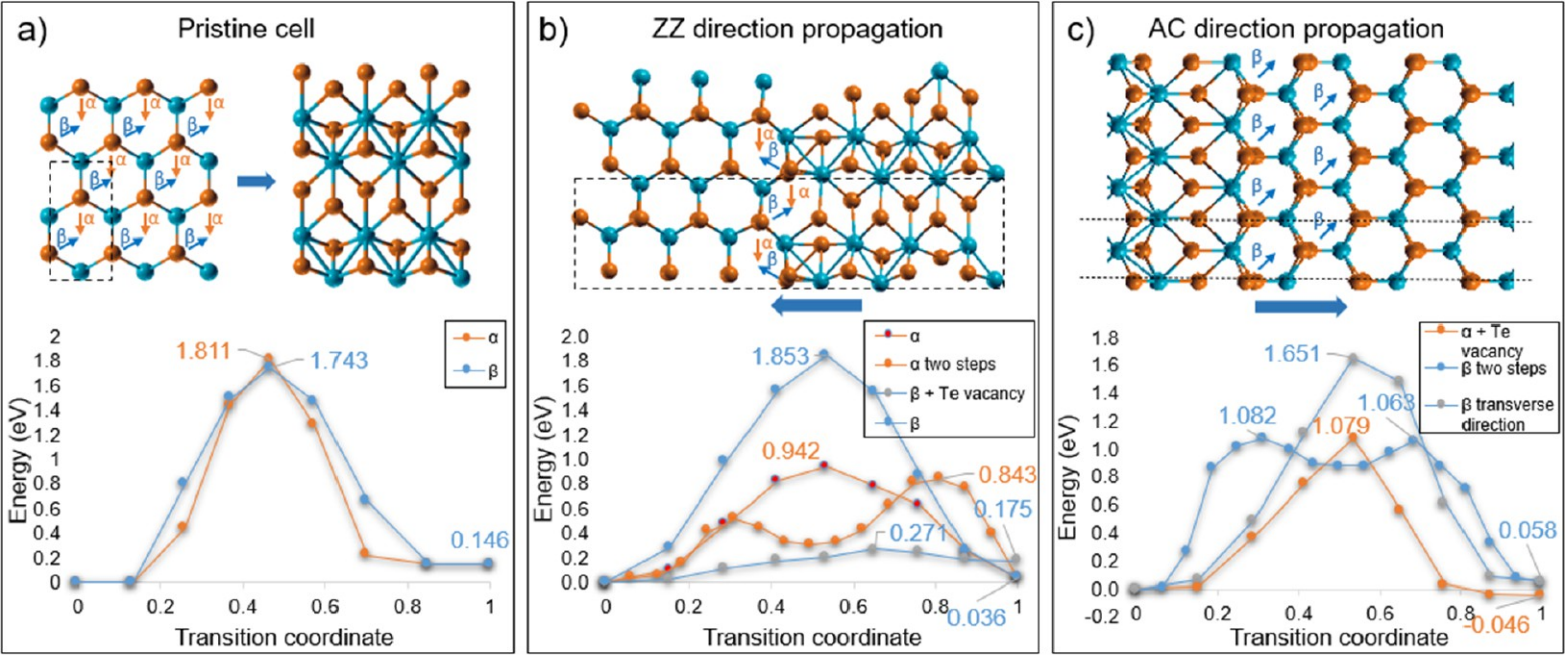
Phase transition energy barriers calculated for pristine case (a) and heterocontact cases with propagation in ZZ (b) and AC (c) directions. Unit cells are indicated by black dashed lines. The total number of atoms used is 6, 41, and 42 for pristine cell, ZZ supercell and AC supercells, respectively.

With the help of first-principles calculations, we now consider the effects of various structural mechanisms on the energetic landscape of single layer MoTe_2_, specifically aiming at assessing the role of the heterocontact and identifying possible structural mechanisms entailing a reduction of the kinetic barrier between 1H and 1T′ phases, thus favoring the phase transition. In the presence of high energy barrier, the 1H to 1T′ transition process might not take place or be too lengthy to be observed. Hence, we performed ab initio simulations of heterocontact-triggered transition paths for the collinear contact (i.e., 1H–ZZ/1T′–ZZ and 1H–AC/1T′–AC) reported in Figure 3d, considering transition paths involving Te atom displacements. First, we calculated energy barriers for pristine cases, which we refer to as α- and β-paths in Figure 6a. Both paths result in the same final 1T′ configuration but originate from the different lower Te atom displacement directions in the initial 1H cell. For the α-path, Te atoms displacement is parallel to the final 1T′–AC direction, while for β it is 45° tilted. We calculated 1.81 (905 meV/f.u.) and 1.74 (870 meV/f.u.) eV energy barriers for α- and β-paths, respectively, with final 1T′ energy 0.146 eV (73 meV/f.u.) higher than initial 1H phase due to the fixed (constant) cell condition. These results are in agreement with previously reported values for pristine cells.^22,30^ Next, we performed simulations for direct 1H–1T′ contact in AC and ZZ direction considering supercells of 7 pristine unit cells (see Figure 6b,c). We refer to the same α and β notations corresponding to the lower Te atoms displacement. We consider broken periodicity in *x* and *y* directions that also results in possible two steps transformations (see Figures S14 and S15) and additional β-path in AC direction corresponding to the transverse direction propagation (see Figure S15) similar to reported in.^28^ Both ZZ and AC routes show similar behavior of α-ZZ, two step α-ZZ and β-AC paths with 0.94 eV (470 meV/f.u.), 0.84 eV (420 meV/f.u.), and 1.08 eV (540 meV/f.u.) energy barriers, respectively. The energy differences between the two phases are 0.036 (18 meV/f.u.) and 0.056 (28 meV/f.u.) eV for the ZZ and AC cases, respectively. We then extended the analysis to defective paths, considering Te vacancy presence at the heterointerface (see Figures S14 and S15). In general, paths that involve Te vacancies are the most promising: in the ZZ case, the presence of a Te vacancy is found to strongly suppress the kinetic barrier between the two phases from 0.94/0.84 eV (470/420 meV/f.u.) in the pristine case to 0.27 eV (135 meV/f.u.). However, the 1T′ phase becomes more unfavorable +0.170 eV (+85 meV/f.u. with respect to the 1H phase. Conversely, in the AC case the energy barrier is not suppressed but the 1T′ becomes energetically favorable at −0.046 eV (−23 meV/f.u.) with respect to the reference 1H phase. These findings are not surprising and consistent with the well-studied Te vacancy induced phase transition mechanism.^27,39^ Our simulations suggest that Te vacancies work differently in AC and ZZ directions and are not the sole responsible for the observed PT; however, they importantly modify the energetic landscape and can be involved in the stabilization of the 1T′ phase in combination with other mechanisms. Reported reduced energy barrier values and lowered ground state energy difference between 1H and 1T′ phases indeed can be responsible for the avalanche effect observed experimentally. However, in the ZZ case, Te vacancies, even with a reduced barrier value, lead to an ascending steps energy profile upon increase of the transformed unit cells number and should result in the backpropagation of 1T′ to 1H transformation of the triggering 1T′ crystal, which we never observe experimentally. In addition, Te vacancies (in both the AC and ZZ directions) tend to accumulate upon phase transition propagation and at some point terminate the transformation (see Figure S14). Therefore, additional mechanisms for 1T′ phase stabilization should be considered such as temperature, higher strain due to the interaction with SiO_2_ substrate or Na and O adatoms^68^ from the initial Na_2_MoO_4_ precursor^66^ (see Supporting Information for discussion).

**Figure 7:**
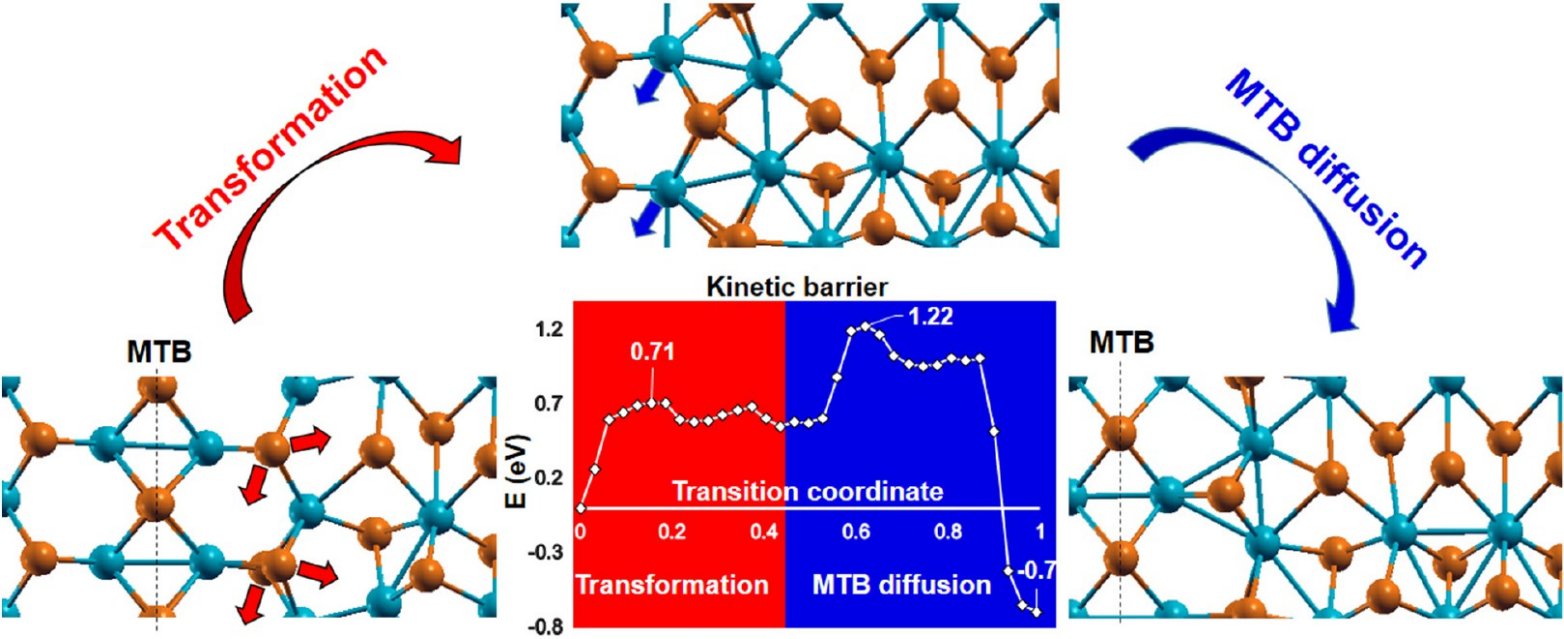
Transformation-diffusion model of the orthogonal b_1T′_/2a_1H_ interface heterocontact. The total number of atoms is 48.

Our DFT simulations also confirm that a heterocontact-triggered phase transition is possible for the orthogonal 1T′–AC/1H–ZZ contact, indicated by the arrow in Figure 3c. In that case, we would expect a huge lattice mismatch up to 10% at the b_1T′_/2a_1H_ interface with approximately 5% 1H–ZZ compressive strain taking place. Raman spectroscopy measurements reveal that the R values of both contacting 1T′ flake and phase transformed flakes are equivalent (or 60° rotated), and hence during this transformation the hexagon’s recrystallization of the 1H–AC into the 1T′–ZZ direction takes place. The possible mechanism of this polymorphism can be explained with a transformation-diffusion model (Figure 7). The initial lowest energy configuration of the heterocontact contains an MTB line and the following Te atom displacement results in the formation of an unstable 1T′ cell with 0.71 eV (355 meV/f.u.) step-like energy barrier, which would make this transformation unfavorable. However, subsequent Mo atoms diffusion (which was shown experimentally at 250 °C^12^ and normally takes place during CVD growth^66^) out of the heterocontact area stabilizes the 1T′ phase with 0.7 eV (350 meV/f.u.) energy gain and final 1.22 eV (610 meV/f.u.) energy barrier.

Finally, two possibilities are represented by other kinds of defects, namely, MTBs and Mo diffusion, since we have observed MTBs and the consequences of Mo diffusion in our grown crystals (e.g., material accumulation and 1T′ post-transformation growth continuation). Thus, we performed additional DFT simulations to understand how these defects affect the transformation. We find that MTBs and Mo excess in ZZ direction generally lead to energetically unfavorable kinetic paths in all the studied situations (see Figures S16 and S17, respectively). More interesting is the case of excess Mo for phase transition propagation in the AC direction. We find intermediate states with double Mo chains formation, resembling the structures reported in monolayer M_4_X_6_ TMDs.^69^ These structures are 0.7–0.77 eV (350–385 meV/f.u.) more favorable than the following 1H–1T′ cell transformation (see Figure S18). In the end, our calculations show a 0.54 eV diffusion barrier in 1H phase via substitutional mechanism in agreement with,^65^ and 0.66 eV in 1T′ phase along the ZZ direction via interstitial diffusion mechanism (see Figure S19). These barriers are significantly lower than those reported for most transition routes. Therefore, we do not exclude that transition kinetics could be limited by Mo diffusion within both the 1H and 1T′ domains and can explain why we observe uncompleted PT for different 1H–1T′ heterocontact angles. Considering few-layer MoTe_2_ formation at the 1H–1T′ heterointerface and 1T′ post-transformation growth continuation, we suppose that the diffusion of excess Mo during CVD growth plays an important role and 1H to 1T′ transformation can be stopped at high Mo concentration or in the presence of defects hindering Mo diffusion.

## Conclusions

In conclusion, we report a semiconductor to semimetal phase transition realized in monolayer MoTe_2_ during CVD growth and triggered by the presence of a direct contact between 1H and 1T′ single crystals. The temperature observed for the phase transition is 730 °C, lower than the temperature reported in^10^ for the annealing of an 1H-MoTe_2_ crystal. We systematically studied the heterocontact-triggered transformation and found that its occurrence and size are independent of the original contact angle and can yield sizable 1H/1T′ lateral heterostructures. The crystal orientation of the transformed 1T′ domains was investigated using linearly polarized Raman spectroscopy and revealed the possibility of two preferential crystal transformation routes: collinear and orthogonal to the initial 1H crystal, both resulting in domains with 60° periodicity. Chemical and structural analyses were performed via XPS and TEM on encapsulated samples to limit their degradation and indicated the presence of MTB and Te vacancies in the 1H crystals. We theoretically modeled transition pathways considering both stoichiometric and nonstoichiometric cases and identified transformation routes with a wide variety of kinetic barrier values. Our simulations demonstrate a significant reduction in the kinetic energy barrier in the heterocontact case compared to the pristine case, which can explain the observed avalanche effect in transformation propagation. Ab initio calculations support the observed phenomenology by indicating that defects such as Te-vacancies favor phase transition, while MTB presence or excess Mo prevent it. For the orthogonal heterocontact case, we propose a transformation–diffusion model involving MTBs to describe the observed polymorphism. The pathways studied in this work can be further investigated in other TMDs, their heterojunctions and in Janus materials.^52^ A recent work has demonstrated that few-layer 2H/1T′ MoTe_2_ heterocontacts can be fabricated through photolithographic techniques, an approach that has been used to recover damaged 2H-MoTe_2_.^70^ While material postprocessing offers enticing prospects for heterocontact formation, in monolayer MoTe_2_ this strategy might be difficult to implement due to the extreme material instability. Defining pathways for bottom-up synthesis of low contact resistance heterojunctions is indeed extremely appealing for fabrication of 2D electronic and optoelectronic devices such as FETs and photodetectors (see Supporting Information for a discussion) and for the development of novel spintronic and quantum devices. By demonstrating heterocontact-induced phase transition and contributing to unveiling the mechanism behind it via theoretical modeling, our work makes a step forward toward the identification of novel strategies for the development of MoTe_2_-based technology.

## Supporting Information Available

Discussion on the 1T′ crystal orientation analysis and determination of the formed 1T′ domains inside 1H hexagons, example of the post-transformation 1T′ growth, description of the Mo:Te ratio extraction approach from the μXPS data, additional KPFM, HRTEM, SAED, and HAADF characterization data, discussion on the transition temperature and chemical stabilization, discussion on the 1H–1T′ heterostructures application, theoretical strain analysis by means of DFT and linear strain theory, details on the DFT simulation of the phase transition propagation for pristine and defective cases and Mo diffusion simulations in both 1H and 1T′ phases of Mo (PDF)

## References

[ref1] Rehn D. A.; Li Y.; Pop E.; Reed E. J. Theoretical Potential for Low Energy Consumption Phase Change Memory Utilizing Electrostatically-Induced Structural Phase Transitions in 2D Materials. Npj Comput. Mater. 2018, 4 (1), 2 10.1038/s41524-017-0059-2.

[ref2] Tang Q. Tuning the Phase Stability of Mo-Based TMD Monolayers through Coupled Vacancy Defects and Lattice Strain. J. Mater. Chem. C 2018, 6 (35), 9561–9568. 10.1039/C8TC03430C.

[ref3] Zhang C.; KC S.; Nie Y.; Liang C.; Vandenberghe W. G.; Longo R. C.; Zheng Y.; Kong F.; Hong S.; Wallace R. M.; Cho K. Charge Mediated Reversible Metal-Insulator Transition in Monolayer MoTe2 and WxMo1-XTe2 Alloy. ACS Nano 2016, 10 (8), 7370–7375. 10.1021/acsnano.6b00148. 27415610

[ref4] Ma R.; Zhang H.; Yoo Y.; Degregorio Z. P.; Jin L.; Golani P.; Ghasemi Azadani J.; Low T.; Johns J. E.; Bendersky L. A.; Davydov A. V.; Koester S. J. MoTe2 Lateral Homojunction Field-Effect Transistors Fabricated Using Flux-Controlled Phase Engineering. ACS Nano 2019, 13 (7), 8035–8046. 10.1021/acsnano.9b02785. 31247141

[ref5] Ruppert C.; Aslan B.; Heinz T. F. Optical Properties and Band Gap of Single- and Few-Layer MoTe _2_ Crystals. Nano Lett. 2014, 14 (11), 6231–6236. 10.1021/nl502557g. 25302768

[ref6] Reyes-Retana J. A.; Cervantes-Sodi F. Spin-Orbital Effects in Metal-Dichalcogenide Semiconducting Monolayers. Sci. Rep. 2016, 6 (1), 24093 10.1038/srep24093. 27094967 PMC4837337

[ref7] Rhodes D. A.; Jindal A.; Yuan N. F. Q.; Jung Y.; Antony A.; Wang H.; Kim B.; Chiu Y.; Taniguchi T.; Watanabe K.; Barmak K.; Balicas L.; Dean C. R.; Qian X.; Fu L.; Pasupathy A. N.; Hone J. Enhanced Superconductivity in Monolayer Td-MoTe2. Nano Lett. 2021, 21 (6), 2505–2511. 10.1021/acs.nanolett.0c04935. 33689385

[ref8] Qian X.; Liu J.; Fu L.; Li J. Quantum Spin Hall Effect in Two-Dimensional Transition Metal Dichalcogenides. Science 2014, 346 (6215), 1344–1347. 10.1126/science.1256815. 25504715

[ref9] Vila M.; Hsu C.-H.; Garcia J. H.; Benítez L. A.; Waintal X.; Valenzuela S. O.; Pereira V. M.; Roche S. Low-Symmetry Topological Materials for Large Charge-to-Spin Interconversion: The Case of Transition Metal Dichalcogenide Monolayers. Phys. Rev. Res. 2021, 3 (4), 043230 10.1103/PhysRevResearch.3.043230.

[ref10] Ryu H.; Lee Y.; Kim H.; Kang S.; Kang Y.; Kim K.; Kim J.; Janicek B. E.; Watanabe K.; Taniguchi T.; Huang P. Y.; Cheong H.; Jung I.; Kim K.; Son Y.; Lee G. Anomalous Dimensionality-Driven Phase Transition of MoTe _2_ in Van Der Waals Heterostructure. Adv. Funct. Mater. 2021, 31 (51), 2107376 10.1002/adfm.202107376.

[ref11] Zhang F.; Zhang H.; Krylyuk S.; Milligan C. A.; Zhu Y.; Zemlyanov D. Y.; Bendersky L. A.; Burton B. P.; Davydov A. V.; Appenzeller J. Electric-Field Induced Structural Transition in Vertical MoTe2- and Mo1-XWxTe2-Based Resistive Memories. Nat. Mater. 2019, 18 (1), 55–61. 10.1038/s41563-018-0234-y. 30542093

[ref12] Zhu H.; Wang Q.; Cheng L.; Addou R.; Kim J.; Kim M. J.; Wallace R. M. Defects and Surface Structural Stability of MoTe _2_ Under Vacuum Annealing. ACS Nano 2017, 11 (11), 11005–11014. 10.1021/acsnano.7b04984. 29116754

[ref13] Guo H.; Yang T.; Yamamoto M.; Zhou L.; Ishikawa R.; Ueno K.; Tsukagoshi K.; Zhang Z.; Dresselhaus M. S.; Saito R. Double Resonance Raman Modes in Monolayer and Few-Layer MoTe 2. Phys. Rev. B 2015, 91 (20), 205415 10.1103/PhysRevB.91.205415.

[ref14] He R.; Zhong S.; Kim H. H.; Ye G.; Ye Z.; Winford L.; McHaffie D.; Rilak I.; Chen F.; Luo X.; Sun Y.; Tsen A. W. Dimensionality-Driven Orthorhombic MoT e 2 at Room Temperature. Phys. Rev. B 2018, 97 (4), 041410 10.1103/PhysRevB.97.041410.

[ref15] Empante T. A.; Zhou Y.; Klee V.; Nguyen A. E.; Lu I.-H.; Valentin M. D.; Naghibi Alvillar S. A.; Preciado E.; Berges A. J.; Merida C. S.; Gomez M.; Bobek S.; Isarraraz M.; Reed E. J.; Bartels L. Chemical Vapor Deposition Growth of Few-Layer MoTe2 in the 2H, 1T′, and 1T Phases: Tunable Properties of MoTe2 Films. ACS Nano 2017, 11 (1), 900–905. 10.1021/acsnano.6b07499. 27992719

[ref16] Tsipas P.; Fragkos S.; Tsoutsou D.; Alvarez C.; Sant R.; Renaud G.; Okuno H.; Dimoulas A. Direct Observation at Room Temperature of the Orthorhombic Weyl Semimetal Phase in Thin Epitaxial MoTe _2_. Adv. Funct. Mater. 2018, 28 (33), 1802084 10.1002/adfm.201802084.

[ref17] Yang D.; Hu X.; Zhuang M.; Ding Y.; Zhou S.; Li A.; Yu Y.; Li H.; Luo Z.; Gan L.; Zhai T. Inversion Symmetry Broken 2D 3R-MoTe2. Adv. Funct. Mater. 2018, 28 (26), 1800785 10.1002/adfm.201800785.

[ref18] Zhang J.; Xia Y.; Wang B.; Jin Y.; Tian H.; Ho W.; Xu H.; Jin C.; Xie M. Single-Layer Mo$\less$sub$\greater$5$\less$/Sub$\greater$Te$\less$sub$\greater$8$\less$/Sub$\greater$ \rule1em1 pt A New Polymorph of Layered Transition-Metal Chalcogenide. 2D Mater. 2021, 8 (1), 015006 10.1088/2053-1583/abbc60.

[ref19] Kim H.; Johns J. E.; Yoo Y. Mixed-Dimensional In-Plane Heterostructures from 1D Mo6Te6 and 2D MoTe2 Synthesized by Te-Flux-Controlled Chemical Vapor Deposition. Small 2020, 16 (47), 2002849 10.1002/smll.202002849. 33103352

[ref20] Pace S.; Martini L.; Convertino D.; Keum D. H.; Forti S.; Pezzini S.; Fabbri F.; Mišeikis V.; Coletti C. Synthesis of Large-Scale Monolayer 1T′-MoTe2 and Its Stabilization via Scalable HBN Encapsulation. ACS Nano 2021, 15 (3), 4213–4225. 10.1021/acsnano.0c05936. 33605730 PMC8023802

[ref21] Yang L.; Wu H.; Zhang W.; Chen Z.; Li J.; Lou X.; Xie Z.; Zhu R.; Chang H. Anomalous Oxidation and Its Effect on Electrical Transport Originating from Surface Chemical Instability in Large-Area, Few-Layer 1T′-MoTe _2_ Films. Nanoscale 2018, 10 (42), 19906–19915. 10.1039/C8NR05699D. 30346016

[ref22] Yuan J.; Chen Y.; Xie Y.; Zhang X.; Rao D.; Guo Y.; Yan X.; Feng Y. P.; Cai Y. Squeezed Metallic Droplet with Tunable Kubo Gap and Charge Injection in Transition Metal Dichalcogenides. Proc. Natl. Acad. Sci. U. S. A. 2020, 117 (12), 6362–6369. 10.1073/pnas.1920036117. 32161125 PMC7104306

[ref23] Zhou X.; Shu H.; Li Q.; Liang P.; Cao D.; Chen X. Electron-Injection Driven Phase Transition in Two-Dimensional Transition Metal Dichalcogenides. J. Mater. Chem. C 2020, 8 (13), 4432–4440. 10.1039/C9TC06410A.

[ref24] Li Y.; Duerloo K.-A. N.; Wauson K.; Reed E. J. Structural Semiconductor-to-Semimetal Phase Transition in Two-Dimensional Materials Induced by Electrostatic Gating. Nat. Commun. 2016, 7 (1), 10671 10.1038/ncomms10671. 26868916 PMC4754345

[ref25] Kolobov A. V.; Fons P.; Tominaga J. Electronic Excitation-Induced Semiconductor-to-Metal Transition in Monolayer MoTe 2. Phys. Rev. B 2016, 94 (9), 094114 10.1103/PhysRevB.94.094114.

[ref26] Krishnamoorthy A.; Bassman Oftelie L.; Kalia R. K.; Nakano A.; Shimojo F.; Vashishta P. Semiconductor-Metal Structural Phase Transformation in MoTe _2_ Monolayers by Electronic Excitation. Nanoscale 2018, 10 (6), 2742–2747. 10.1039/C7NR07890K. 29334101

[ref27] Si C.; Choe D.; Xie W.; Wang H.; Sun Z.; Bang J.; Zhang S. Photoinduced Vacancy Ordering and Phase Transition in MoTe _2_. Nano Lett. 2019, 19 (6), 3612–3617. 10.1021/acs.nanolett.9b00613. 31096752

[ref28] Ghasemi A.; Gao W. Atomistic Mechanism of Stress Modulated Phase Transition in Monolayer MoTe 2. Extreme Mech. Lett. 2020, 40, 100946 10.1016/j.eml.2020.100946.

[ref29] Duerloo K.-A. N.; Li Y.; Reed E. J. Structural Phase Transitions in Two-Dimensional Mo- and W-Dichalcogenide Monolayers. Nat. Commun. 2014, 5 (1), 4214 10.1038/ncomms5214. 24981779

[ref30] Huang H. H.; Fan X.; Singh D. J.; Chen H.; Jiang Q.; Zheng W. T. Controlling Phase Transition for Single-Layer MTe _2_ (M = Mo and W): Modulation of the Potential Barrier under Strain. Phys. Chem. Chem. Phys. 2016, 18 (5), 4086–4094. 10.1039/C5CP06706E. 26778806

[ref31] Young J.; Reinecke T. L. Controlling the H to T′ Structural Phase Transition via Chalcogen Substitution in MoTe2Monolayers. Phys. Chem. Chem. Phys. 2017, 19 (47), 31874–31882. 10.1039/C7CP05634F. 29177330

[ref32] Manchanda P.; Kumar P.; Dev P. Thickness Dependence of Hydrogen-Induced Phase Transition in MoTe 2. Phys. Rev. B 2020, 101 (14), 144104 10.1103/PhysRevB.101.144104.

[ref33] Vellinga M. B.; de Jonge R.; Haas C. Semiconductor to Metal Transition in MoTe2. J. Solid State Chem. 1970, 2 (2), 299–302. 10.1016/0022-4596(70)90085-X.

[ref34] Zakhidov D.; Rehn D. A.; Reed E. J.; Salleo A. Reversible Electrochemical Phase Change in Monolayer to Bulk-like MoTe _2_ by Ionic Liquid Gating. ACS Nano 2020, 14 (3), 2894–2903. 10.1021/acsnano.9b07095. 32045212

[ref35] Wang Y.; Zhang M.; Xue Z.; Chen X.; Mei Y.; Chu P. K.; Tian Z.; Wu X.; Di Z. Atomistic Observation of the Local Phase Transition in MoTe _2_ for Application in Homojunction Photodetectors. Small 2022, 18 (19), 2200913 10.1002/smll.202200913. 35411673

[ref36] Eshete Y. A.; Ling N.; Kim S.; Kim D.; Hwang G.; Cho S.; Yang H. Vertical Heterophase for Electrical, Electrochemical, and Mechanical Manipulations of Layered MoTe _2_. Adv. Funct. Mater. 2019, 29 (40), 1904504 10.1002/adfm.201904504.

[ref37] Wang Y.; Xiao J.; Zhu H.; Li Y.; Alsaid Y.; Fong K. Y.; Zhou Y.; Wang S.; Shi W.; Wang Y.; Zettl A.; Reed E. J.; Zhang X. Structural Phase Transition in Monolayer MoTe2 Driven by Electrostatic Doping. Nature 2017, 550 (7677), 487–491. 10.1038/nature24043. 29019982

[ref38] Shi J.; Bie Y.-Q.; Zong A.; Fang S.; Chen W.; Han J.; Cao Z.; Zhang Y.; Taniguchi T.; Watanabe K.; Bulović V.; Kaxiras E.; Baldini E.; Jarillo-Herrero P.; Nelson K. A. Intrinsic 1T’ Phase Induced in Atomically Thin 2H-MoTe_2_ by a Single Terahertz Pulse. arXiv 2019 (https://doi.org/10.48550/ARXIV.1910.13609). 10.1038/s41467-023-41291-w PMC10516973 37737233

[ref39] Köster J.; Ghorbani-Asl M.; Komsa H.-P.; Lehnert T.; Kretschmer S.; Krasheninnikov A. V.; Kaiser U. Defect Agglomeration and Electron-Beam-Induced Local-Phase Transformations in Single-Layer MoTe _2_. J. Phys. Chem. C 2021, 125 (24), 13601–13609. 10.1021/acs.jpcc.1c02202.

[ref40] Ripoll-Sau J.; Calleja F.; Casado Aguilar P.; Ibarburu I. M.; Vázquez de Parga A. L.; Miranda R.; Garnica M. Phase Control and Lateral Heterostructures of MoTe _2_ Epitaxially Grown on Graphene/Ir(111). Nanoscale 2022, 14 (30), 10880–10888. 10.1039/D2NR03074H. 35848284

[ref41] Keum D. H.; Cho S.; Kim J. H.; Choe D.-H.; Sung H.-J.; Kan M.; Kang H.; Hwang J.-Y.; Kim S. W.; Yang H.; Chang K. J.; Lee Y. H. Bandgap Opening in Few-Layered Monoclinic MoTe2. Nat. Phys. 2015, 11 (6), 482–486. 10.1038/nphys3314.

[ref42] Ueno K.; Fukushima K. Changes in Structure and Chemical Composition of α-MoTe _2_ and β-MoTe _2_ during Heating in Vacuum Conditions. Appl. Phys. Express 2015, 8 (9), 095201 10.7567/APEX.8.095201.

[ref43] Miseikis V.; Bianco F.; David J.; Gemmi M.; Pellegrini V.; Romagnoli M.; Coletti C. Deterministic Patterned Growth of High-Mobility Large-Crystal Graphene: A Path towards Wafer Scale Integration. 2D Mater. 2017, 4 (2), 021004 10.1088/2053-1583/aa5481.

[ref44] Churchill H. O. H.; Salamo G. J.; Yu S.-Q.; Hironaka T.; Hu X.; Stacy J.; Shih I. Toward Single Atom Chains with Exfoliated Tellurium. Nanoscale Res. Lett. 2017, 12 (1), 488 10.1186/s11671-017-2255-x. 28799071 PMC5552621

[ref45] Khatun S.; Banerjee A.; Pal A. J. Nonlayered Tellurene as an Elemental 2D Topological Insulator: Experimental Evidence from Scanning Tunneling Spectroscopy. Nanoscale 2019, 11 (8), 3591–3598. 10.1039/C8NR09760G. 30734805

[ref46] Linck M.; Hartel P.; Uhlemann S.; Kahl F.; Müller H.; Zach J.; Haider M.; Niestadt M.; Bischoff M.; Biskupek J.; Lee Z.; Lehnert T.; Börrnert F.; Rose H.; Kaiser U. Chromatic Aberration Correction for Atomic Resolution TEM Imaging from 20 to 80 KV. Phys. Rev. Lett. 2016, 117 (7), 076101 10.1103/PhysRevLett.117.076101. 27563976

[ref47] Giannozzi P.; Baroni S.; Bonini N.; Calandra M.; Car R.; Cavazzoni C.; Ceresoli D.; Chiarotti G. L.; Cococcioni M.; Dabo I.; Dal Corso A.; de Gironcoli S.; Fabris S.; Fratesi G.; Gebauer R.; Gerstmann U.; Gougoussis C.; Kokalj A.; Lazzeri M.; Martin-Samos L.; Marzari N.; Mauri F.; Mazzarello R.; Paolini S.; Pasquarello A.; Paulatto L.; Sbraccia C.; Scandolo S.; Sclauzero G.; Seitsonen A. P.; Smogunov A.; Umari P.; Wentzcovitch R. M. QUANTUM ESPRESSO: A Modular and Open-Source Software Project for Quantum Simulations of Materials. J. Phys.: Condens. Matter 2009, 21 (39), 395502 10.1088/0953-8984/21/39/395502. 21832390

[ref48] Prandini G.; Marrazzo A.; Castelli I. E.; Mounet N.; Marzari N. Precision and Efficiency in Solid-State Pseudopotential Calculations. Npj Comput. Mater. 2018, 4 (1), 1–13. 10.1038/s41524-018-0127-2.

[ref49] Schlipf M.; Gygi F. Optimization Algorithm for the Generation of ONCV Pseudopotentials. Comput. Phys. Commun. 2015, 196, 36–44. 10.1016/j.cpc.2015.05.011.

[ref50] Garrity K. F.; Bennett J. W.; Rabe K. M.; Vanderbilt D. Pseudopotentials for High-Throughput DFT Calculations. Comput. Mater. Sci. 2014, 81, 446–452. 10.1016/j.commatsci.2013.08.053.

[ref51] Marzari N.; Vanderbilt D.; De Vita A.; Payne M. C. Thermal Contraction and Disordering of the Al(110) Surface. Phys. Rev. Lett. 1999, 82 (16), 3296–3299. 10.1103/PhysRevLett.82.3296.

[ref52] Yagmurcukardes M.; Sevik C.; Peeters F. M. Electronic, Vibrational, Elastic, and Piezoelectric Properties of Monolayer Janus MoSTe Phases: A First-Principles Study. Phys. Rev. B 2019, 100 (4), 045415 10.1103/PhysRevB.100.045415.

[ref53] Cadelano E.; Palla P. L.; Giordano S.; Colombo L. Elastic Properties of Hydrogenated Graphene. Phys. Rev. B 2010, 82 (23), 235414 10.1103/PhysRevB.82.235414.

[ref54] Kan M.; Nam H. G.; Lee Y. H.; Sun Q. Phase Stability and Raman Vibration of the Molybdenum Ditelluride (MoTe _2_) Monolayer. Phys. Chem. Chem. Phys. 2015, 17 (22), 14866–14871. 10.1039/C5CP01649E. 25982102

[ref55] Grzeszczyk M.; Gołasa K.; Molas M. R.; Nogajewski K.; Zinkiewicz M.; Potemski M.; Wysmołek A.; Babiński A. Raman Scattering from the Bulk Inactive out-of-Plane $${{\bf{B}}}_{{\bf{2}}{\bf{g}}}^{{\bf{1}}}$$ B 2 g 1 Mode in Few-Layer MoTe2. Sci. Rep. 2018, 8 (1), 17745 10.1038/s41598-018-35510-4. 30531971 PMC6288152

[ref56] Chen S.-Y.; Naylor C. H.; Goldstein T.; Johnson A. T. C.; Yan J. Intrinsic Phonon Bands in High-Quality Monolayer T′ Molybdenum Ditelluride. ACS Nano 2017, 11 (1), 814–820. 10.1021/acsnano.6b07260. 27943667

[ref57] Beams R.; Cançado L. G.; Krylyuk S.; Kalish I.; Kalanyan B.; Singh A. K.; Choudhary K.; Bruma A.; Vora P. M.; Tavazza F.; Davydov A. V.; Stranick S. J. Characterization of Few-Layer 1T′ MoTe2 by Polarization-Resolved Second Harmonic Generation and Raman Scattering. ACS Nano 2016, 10 (10), 9626–9636. 10.1021/acsnano.6b05127. 27704774 PMC5542881

[ref58] Zhou L.; Huang S.; Tatsumi Y.; Wu L.; Guo H.; Bie Y.-Q.; Ueno K.; Yang T.; Zhu Y.; Kong J.; Saito R.; Dresselhaus M. Sensitive Phonon-Based Probe for Structure Identification of 1T′ MoTe2. J. Am. Chem. Soc. 2017, 139 (25), 8396–8399. 10.1021/jacs.7b03445. 28541698

[ref59] Song Q.; Wang H.; Pan X.; Xu X.; Wang Y.; Li Y.; Song F.; Wan X.; Ye Y.; Dai L. Anomalous In-Plane Anisotropic Raman Response of Monoclinic Semimetal 1 T́-MoTe 2. Sci. Rep. 2017, 7 (1), 1758 10.1038/s41598-017-01874-2. 28496170 PMC5431984

[ref60] Hoang A. T.; Shinde S. M.; Katiyar A. K.; Dhakal K. P.; Chen X.; Kim H.; Lee S. W.; Lee Z.; Ahn J.-H. Orientation-Dependent Optical Characterization of Atomically Thin Transition Metal Ditellurides. Nanoscale 2018, 10 (46), 21978–21984. 10.1039/C8NR07592A. 30451270

[ref61] Wang J.; Luo X.; Li S.; Verzhbitskiy I.; Zhao W.; Wang S.; Quek S. Y.; Eda G. Determination of Crystal Axes in Semimetallic T′-MoTe2 by Polarized Raman Spectroscopy. Adv. Funct. Mater. 2017, 27 (14), 1604799 10.1002/adfm.201604799.

[ref62] Berry J.; Zhou S.; Han J.; Srolovitz D. J.; Haataja M. P. Domain Morphology and Mechanics of the H/T ′ Transition Metal Dichalcogenide Monolayers. Phys. Rev. Mater. 2018, 2 (11), 114002 10.1103/PhysRevMaterials.2.114002.

[ref63] Han G. H.; Keum D. H.; Zhao J.; Shin B. G.; Song S.; Bae J. J.; Lee J.; Kim J. H.; Kim H.; Moon B. H.; Lee Y. H. Absorption Dichroism of Monolayer 1T′-MoTe2 in Visible Range. 2D Mater. 2016, 3 (3), 031010 10.1088/2053-1583/3/3/031010.

[ref64] Mc Manus J. B.; Cunningham G.; McEvoy N.; Cullen C. P.; Gity F.; Schmidt M.; McAteer D.; Mullarkey D.; Shvets I. V.; Hurley P. K.; Hallam T.; Duesberg G. S. Growth of 1T′ MoTe2 by Thermally Assisted Conversion of Electrodeposited Tellurium Films. ACS Appl. Energy Mater. 2019, 2 (1), 521–530. 10.1021/acsaem.8b01540.

[ref65] Coelho P. M.; Komsa H.-P.; Coy Diaz H.; Ma Y.; Krasheninnikov A. V.; Batzill M. Post-Synthesis Modifications of Two-Dimensional MoSe2 or MoTe2 by Incorporation of Excess Metal Atoms into the Crystal Structure. ACS Nano 2018, 12 (4), 3975–3984. 10.1021/acsnano.8b01580. 29630829

[ref66] Kim H.; Han G. H.; Yun S. J.; Zhao J.; Keum D. H.; Jeong H. Y.; Ly T. H.; Jin Y.; Park J.-H.; Moon B. H.; Kim S.-W.; Lee Y. H. Role of Alkali Metal Promoter in Enhancing Lateral Growth of Monolayer Transition Metal Dichalcogenides. Nanotechnology 2017, 28 (36), 36LT01 10.1088/1361-6528/aa7e5e. 28686170

[ref67] Wang Z.-Y.; Zhou Y.-L.; Wang X.-Q.; Wang F.; Sun Q.; Guo Z.-X.; Jia Y. Effects of In-Plane Stiffness and Charge Transfer on Thermal Expansion of Monolayer Transition Metal Dichalcogenide*. Chin. Phys. B 2015, 24 (2), 026501 10.1088/1674-1056/24/2/026501.

[ref68] Zhou Y.; Reed E. J. Structural Phase Stability Control of Monolayer MoTe _2_ with Adsorbed Atoms and Molecules. J. Phys. Chem. C 2015, 119 (37), 21674–21680. 10.1021/acs.jpcc.5b05770.

[ref69] Hong J.; Chen X.; Li P.; Koshino M.; Li S.; Xu H.; Hu Z.; Ding F.; Suenaga K. Multiple 2D Phase Transformations in Monolayer Transition Metal Chalcogenides. Adv. Mater. 2022, 34 (19), 2200643 10.1002/adma.202200643. 35307877

[ref70] Xu X.; Han B.; Liu S.; Yang S.; Jia X.; Xu W.; Gao P.; Ye Y.; Dai L. Atomic-Precision Repair of a Few-Layer 2H-MoTe2 Thin Film by Phase Transition and Recrystallization Induced by a Heterophase Interface. Adv. Mater. 2020, 32 (23), 2000236 10.1002/adma.202000236. 32329549

