# Supplementary material for: Heterocontact-Triggered 1H to 1T′ Phase Transition in CVD-Grown Monolayer MoTe2: Implications for Low Contact Resistance Electronic Devices
Source: ACS Appl Nano Mater. 2023 Jul 6;7(16):18094–105. doi: 10.1021/acsanm.3c01314 (PMC11348416; doi:10.1021/acsanm.3c01314)
Supplement: Supplementary file 1 — an3c01314_si_001.pdf [file an3c01314_si_001.pdf]

Supporting Information

Heterocontact-Triggered 1H to 1T' Phase Transition in CVD-Grown Monolayer $MoTe_2$: Implications for Low Contact Resistance Electronic Devices

Vladislav O. Khaustov*[1,2], Domenica Convertino[1], Janis Köster[3], Alexei A. Zakharov[4], Michael J. Mohn[3], Zewdu M. Gebeyehu[1,5], Leonardo Martini[1], Simona Pace[1,5], Giovanni Marini[5], Matteo Calandra[5,6,7], Ute Kaiser[3], Stiven Forti[1], Camilla Coletti*[1,5]

1. Center for Nanotechnology Innovation @NEST, Istituto Italiano di Tecnologia, Piazza San Silvestro 12, I-56127 Pisa, Italy.

2. NEST, Scuola Normale Superiore, Piazza San Silvestro 12, I-56127 Pisa, Italy.

3.Ulm University, Central Facility for Electron Microscopy, Materials Science Electron Microscopy, Albert-Einstein-Allee 11, D-89081 Ulm, Germany

4. MAX IV Laboratory, Lund University, P.O. Box 118, Lund, S-22100, Sweden.

5. Graphene Labs, Istituto Italiano di Tecnologia, Via Morego 30, 16163 Genova, Italy.

6. Department of Physics, University of Trento, Via Sommarive 14, 38123 Povo, Italy

7. Sorbonne Université, CNRS, Institut des Nanosciences de Paris, UMR7588, F-75252 Paris, France

Corresponding authors

Email for V.K.: vladislav.khaustov@sns.it

Email for C.C.: camilla.coletti@iit.it

1. **Growth setup schematic**

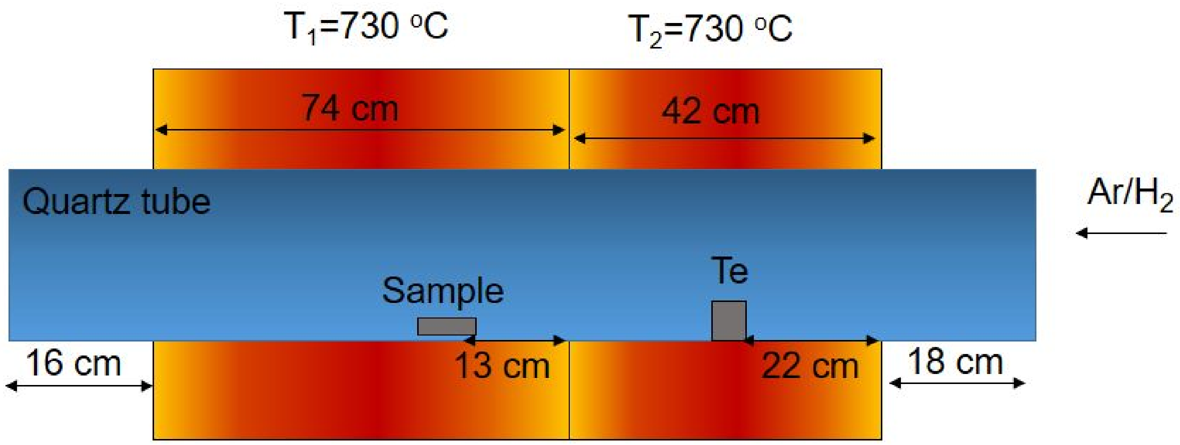

Figure S1. Schematic of the growth setup with relative distances between sample and alumina crucible (Te) indicated.

2. **Growth time variation experiment**

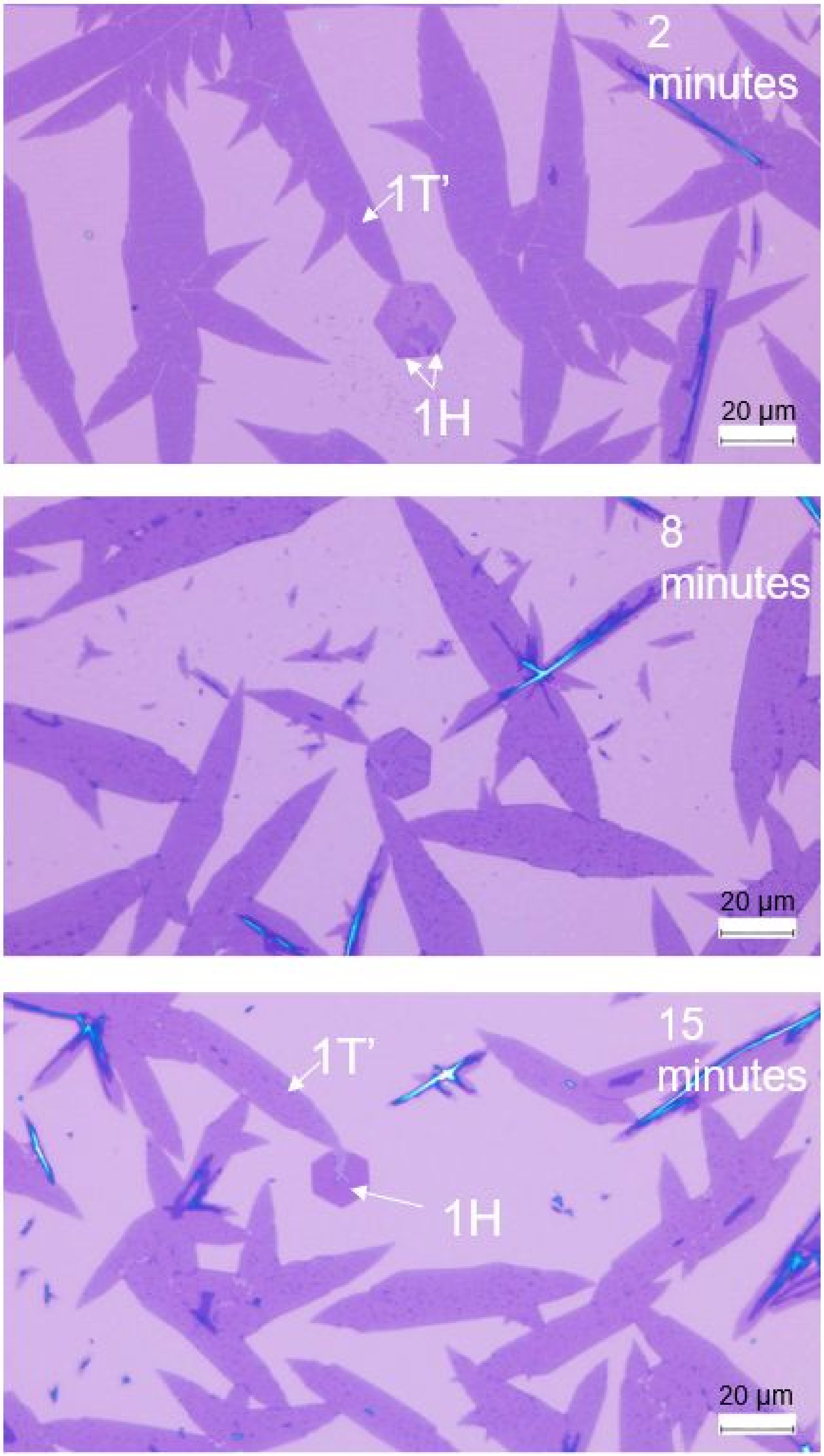

Figure S2. a) Results on the growth time variation. CVD growth as well as 1H to 1T' phase transition demonstrate "explosive" nature. Partially or fully transformed samples are observed regardless of the growth time.

3. **Fully transformed hexagonal flake**

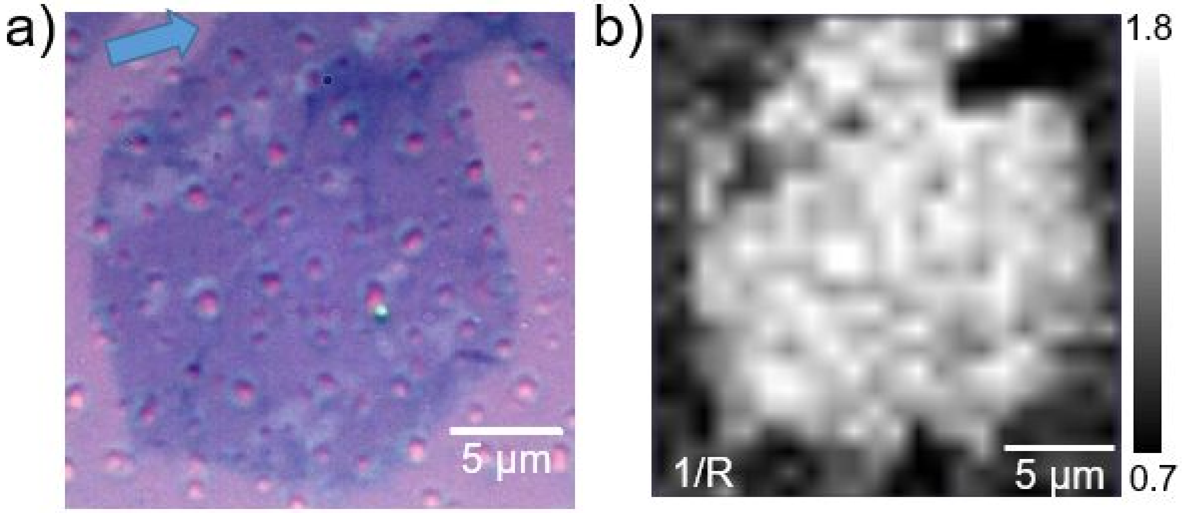

Figure S3. Fully transformed 1T’ hexagon. a) Optical image of the flake in contact with two 1T’ flakes; bright areas are oxidized 1T’ due to the defects in hBN encapsulation layer. b) 1/R-value Raman map (see next section) showing that the hexagonal flake adopted the crystal orientation of the 1T’ elongated flake indicated by the light blue arrow.

4. **Linearly polarized Raman spectroscopy crystal direction identification via sample rotation**

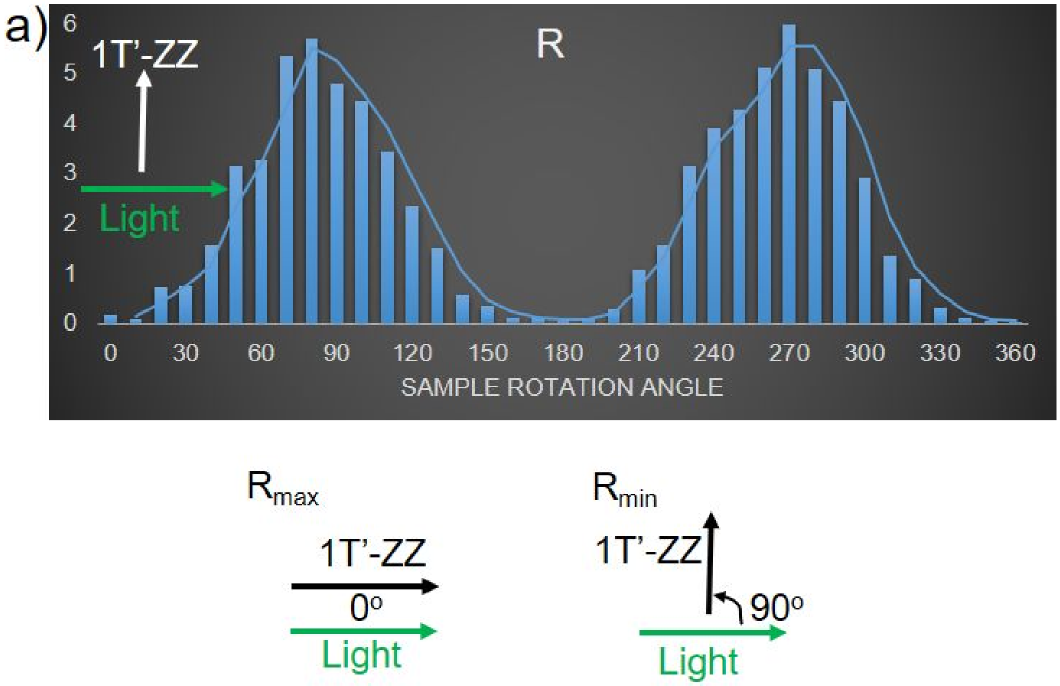

Figure S4. Collected linearly polarized Raman spectroscopy data for R-value on the sample rotation angle. ZZ direction of the 1T’ flake is determined by rotating the crystal and minimizing R-value.

5. 1T’ crystal direction identification.

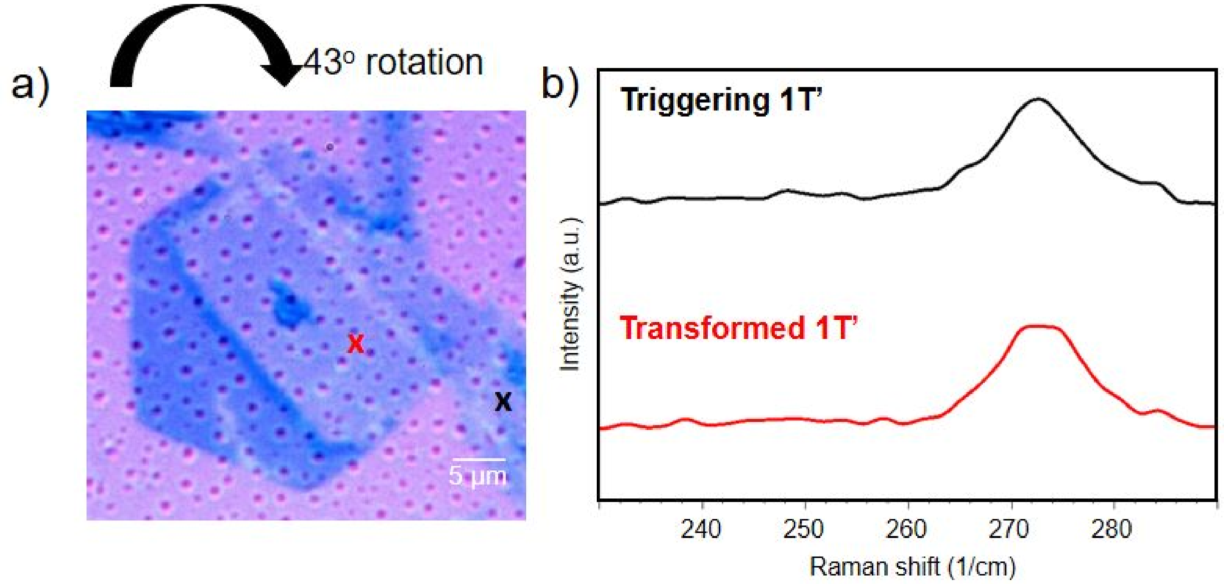

Figure S5. Crystal direction identification of the triggering and transformed 1T’ crystals from Figure 1 of the main text. a) The sample was rotated by 43 degrees in order to minimize R-value for the transformed 1T’ region. b) Single Raman spectra showing equivalent I(253 cm^{-1})/I(271 cm^{-1}) ratio for the transformed 1T’ region and lower 1T’ flake, which confirms that the PT was triggered by the lower contacting flake.

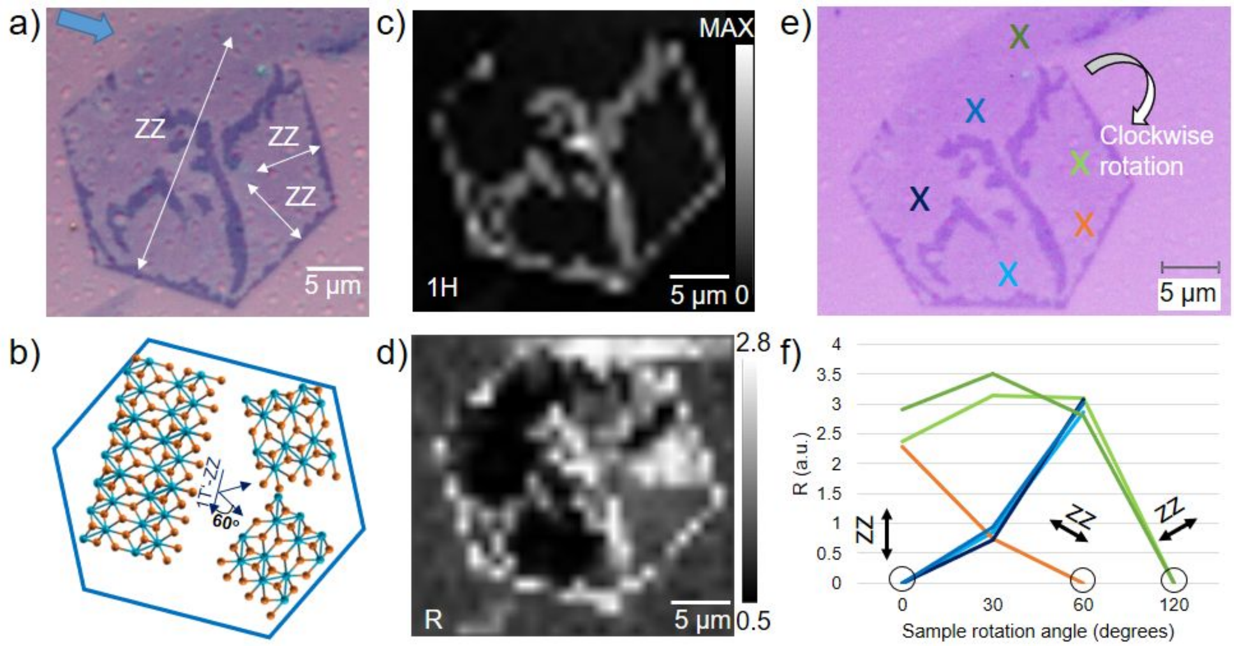

Figure S6. Different crystallographic directions in 1T’ transformed portions of a hexagonal crystal. a) Optical image with white arrows showing assigned 1T’ crystal directions. b) Ball-and-stick model depicting the various crystallographic directions identified in the hexagonal flake for 1T’ phase. c) Raman map of the E^1_{2g} mode of the 1H phase. d) R-value mapping showing three areas with different colors (white, gray and black), hence three different crystal orientations. To identify the crystals directions the sample was rotated clockwise in order to minimize the R-value for each 1T’ domain found. The R-value was measured for the 6 points of different colors in (e) and the results plotted for the different rotation angles from 0 to 120 degrees in (f).

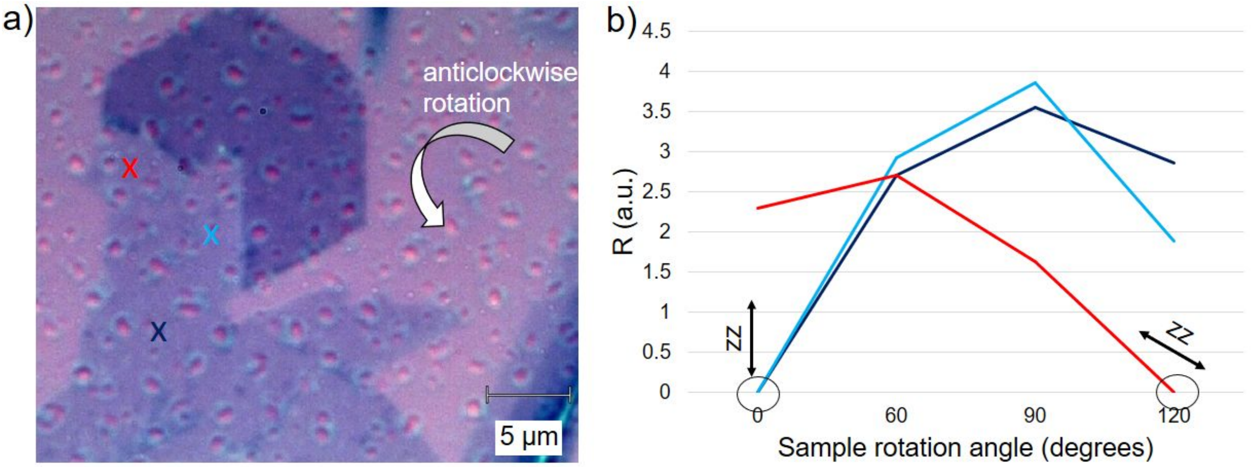

Figure S7. Crystal direction identification of the transformed 1T’ domains from Figure 2 (d, e) of the main text. The sample was rotated anticlockwise in order to minimize R-value for each 1T’ domain found. The R-value was measured in correspondence of the 3 crosses indicated in (a) and the results plotted for the different rotation angles from 0 to 120 degrees in (b).

6. 1T’ crystals post-transformation growth.

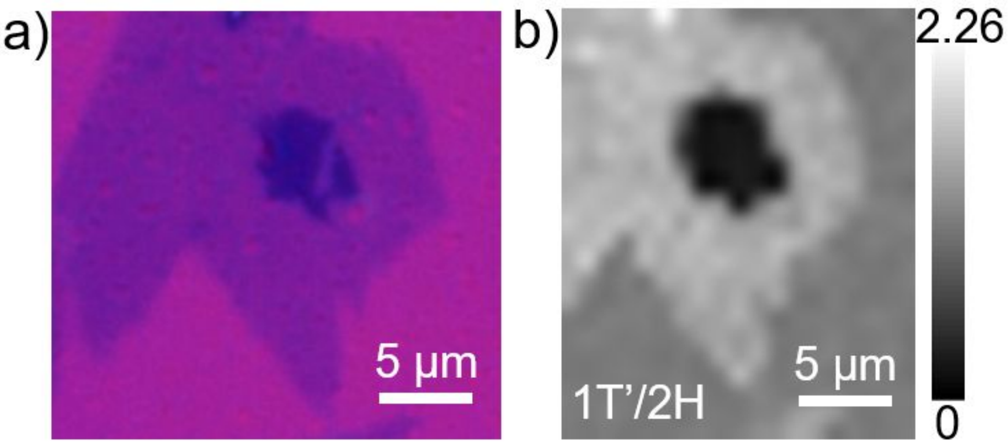

Figure S8. An example of a transformed 1H hexagon, where 1T’ spikes continue to grow out of the hexagon plane after the transformation. a) Optical image. b) Raman 1T’/2H map (84 cm^{-1}/232 cm^{-1})

7. Mo:Te ratio extraction from µXPS measurements.

We performed XPS measurement in a configuration with both Incident photon beam and detected photoelectrons' angles parallel to the surface normal. In this case, the total emission of photoelectrons in a small solid angle ΔΩ for a given subshell *i* for a given element *A* with photoelectron energy *E* obtained from the depth *z* can be described by the following equation [1]:

$$I_{i,\mathrm{A},E} = \frac{\Delta\Omega}{4\pi} I_{hv} \sigma_{i,\ \mathrm{A},E} W_{i,\ \mathrm{A},E}(\beta) G_E N_A \int_0^d e^{\left(-\frac{z}{\lambda_{A,\ E}}\right)} dz, \qquad (1)$$

where I_{hv} is the photon flux, $\sigma_{i,E}$ is the photoionization cross-section, $W_{i,E}$ is the angular asymmetry factor which depends on the asymmetry parameter β, G_E is the analyzer efficiency that is proportional to *1/E*, N_A is the density of *A* atoms, *d* is the sample thickness and $\lambda_{A,E}$ is the attenuation length. Photon energy E_{hv} and photoelectron energy E are 350, 100 eV and 115.5, 54 eV for Mo and Te, respectively. The photoionization cross-section $\sigma_{i,E}$ and asymmetry parameter β are taken from the database [2]. β was replaced by the effective asymmetry parameter β_{eff} suggested for solid matter [1]. β_{eff} and $\lambda_{A,E}$ values were taken from the NIST software, with $\lambda_{A,E}$ calculated by Cumpson-Seah expression (CS2) for each element and given energy E [3]. All constants' values are presented in the Table S1, for simplicity we replace the final product of the constants before integral and $\lambda_{A,E}$ in eq. 1 by constant K_i in the following calculations.

Table S1. XPS parameters

Element/Constant	I_{hv}, ph/s	$\sigma_{i,E}$, Mbarn	β (β_{eff})	$W_{i,E}$	G_E	λ_A(54 eV), A	λ_A(115.5 eV), A
Mo	$83 \cdot 10^{17}$	4.195	0.46(0.3)	0.85	1/115.5	2.65	3.49
Te	$101 \cdot 10^{17}$	4.082	1.64(0.95)	0.53	1/54	3.77	4.88

Solving eq. 1 for both Te and Mo atoms and considering screening effects of the upper layers, we obtain the following equations for Mo and Te intensities, respectively:

$$I_{Mo} = K_{Mo} N_{Mo} X_C X_{Te} \left(\sum_{n=1}^{m} \left(X_{Te}^2 X_{Mo} \right)^{n-1} \right) (1 - X_{Mo}), \qquad (2)$$

$$I_{Te} = K_{Te} N_{Te} X_C \left(1 + X_{Te} X_{Mo} + \sum_{n=2}^{m} \left(X_{Te}^n X_{Mo}^{n-1} + X_{Te}^{n+1} X_{Mo}^n \right) \right) (1 - X_{Te}), \qquad (3)$$

where *n* corresponds to the number of $MoTe_2$ layers and $X_A = e^{\left(-\frac{d_A}{\lambda_{A,\ E}}\right)}$, represents the screening of the upper layers (C, Mo and Te). Inelastic mean free path was reported to be constant in Graphene for 50-100 eV kinetic energies range [4], therefore, we do not expect significant screening effect and neglect X_C in the following calculations. For quantitative analysis we treat monolayer $MoTe_2$ as three separated Te-Mo-Te layers with equal thickness (2.4 A) that corresponds to the 1/3 of the total 7.2 A interlayer spacing. Eq. 2 and 3 allow extracting N_{Mo}/N_{Te} using measured I_{Mo} and I_{Te} for different regions. For experimental few-layer and bulk data analysis, expanding eq. 2 and 3 up to the 3rd term is sufficient, because higher order terms give insignificant contribution. According to the obtained equations, we would expect to see at least 19 % of Mo and 13% of Te intensities increment upon moving from monolayer to bulk regions. However, we observe Te intensity increase while Mo signal remains almost constant and even slightly decreases in the 2H seed region. It can be explained by $2H_c$ symmetry of layered $MoTe_2$ structure, where Mo atoms of the second layer are located directly in line with two upper Te atoms of the first layer (see Figure S9).

Considering our "0 angle" incidence-detection geometry and low energy photon energies, it is not surprising that we can detect Mo signal only from the upper monolayer. In Te case, we still can see signal from the upper Te layer of the second $MoTe_2$ layer because there is only one Mo atom positioned directly above it. In addition, excess Te atoms in the interlayer spacing can be detected. Hence, for 1H-1T' and 2H seed area analysis we use only first layer contribution for Mo intensity and first layer + upper Te layer contribution of the second $MoTe_2$ layer for Te intensity. Mo signal attenuation in the seed area can be attributed to the defects in graphene layer and residual adsorbents contribution, which serves as a contamination layer. It is not trivial to estimate attenuation length for contamination layer without knowledge of thickness and exact composition. Therefore, we assign Mo signal 8.5% difference to the extracted Mo:Te ratio error. Intensities, X_A and final Mo:Te ratio for different regions summarized in the Table S2.

Table S2. Measured Intensities and calculated X_A parameters

Element/Parameter	I, 1H	I, 1H-1T'	I,2H	X_A (54 eV)	X_A (115.5 eV)
Mo	82	81	75	0.405	0.502
Te	449	544	602	0.529	0.611
Mo:Te	0.57±0.05	0.51±0.04	0.43±0.04		

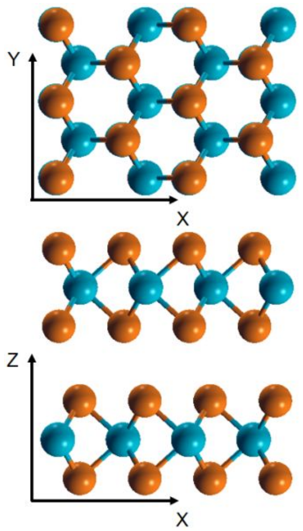

Figure S9. Planar (top) and side (bottom) views of the layered $2H_c$-$MoTe_2$ structure.

8. HRTEM characterization.

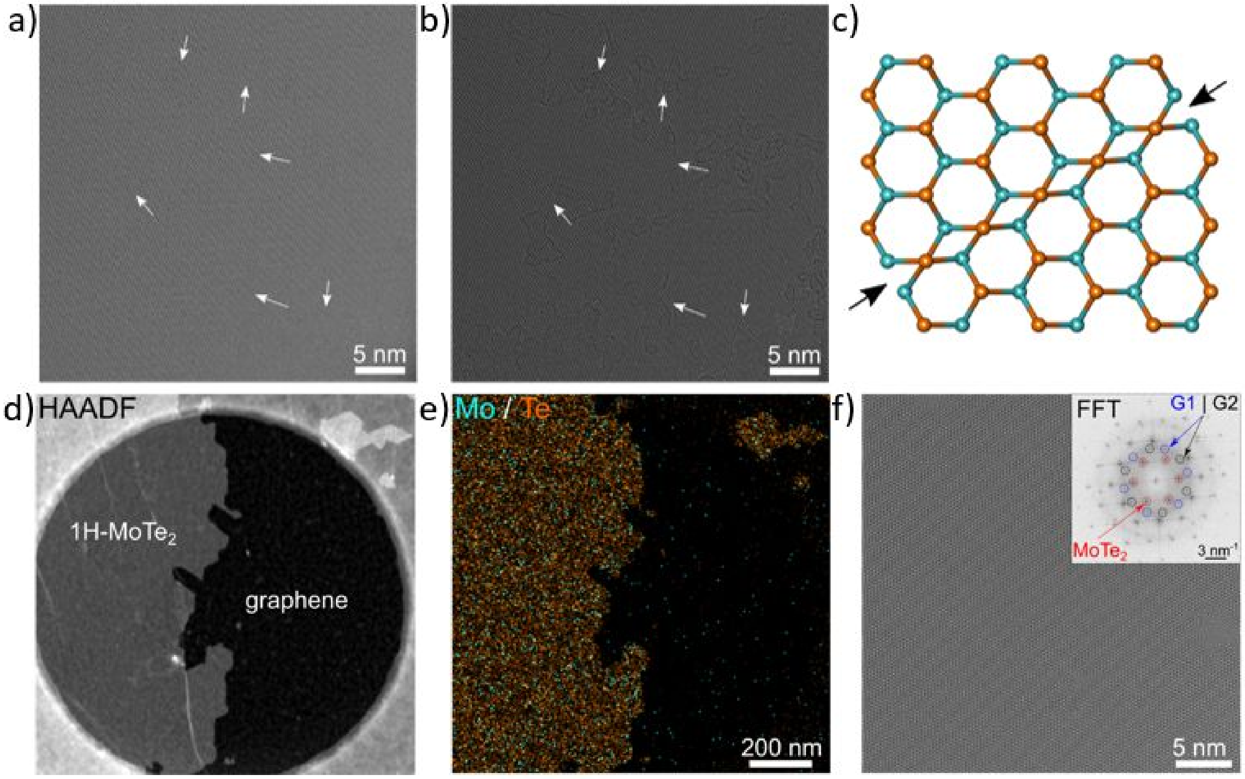

Figure S10. a) HRTEM image of monolayer 1H-$MoTe_2$ with 4|4P MTB domains. White arrows mark kinks in the MTBs. b) defocused image of a) for better visibility of the MTBs. c) structure model of the 4|4P MTB domain structure. (d-f) (S)TEM characterization of transferred 1H-$MoTe_2$. d) STEM 80 kV HAADF image of the suspended flake, showing HC area where 1T' did not survive the transfer. e) EDX elemental map characterization of the same region. Molybdenum is colored cyan and tellurium orange. f) 80 kV HRTEM image of 1H phase and corresponding FFT, first order 1H-$MoTe_2$ and graphene (G) spots are highlighted by red and blue, black dotted rings, respectively.

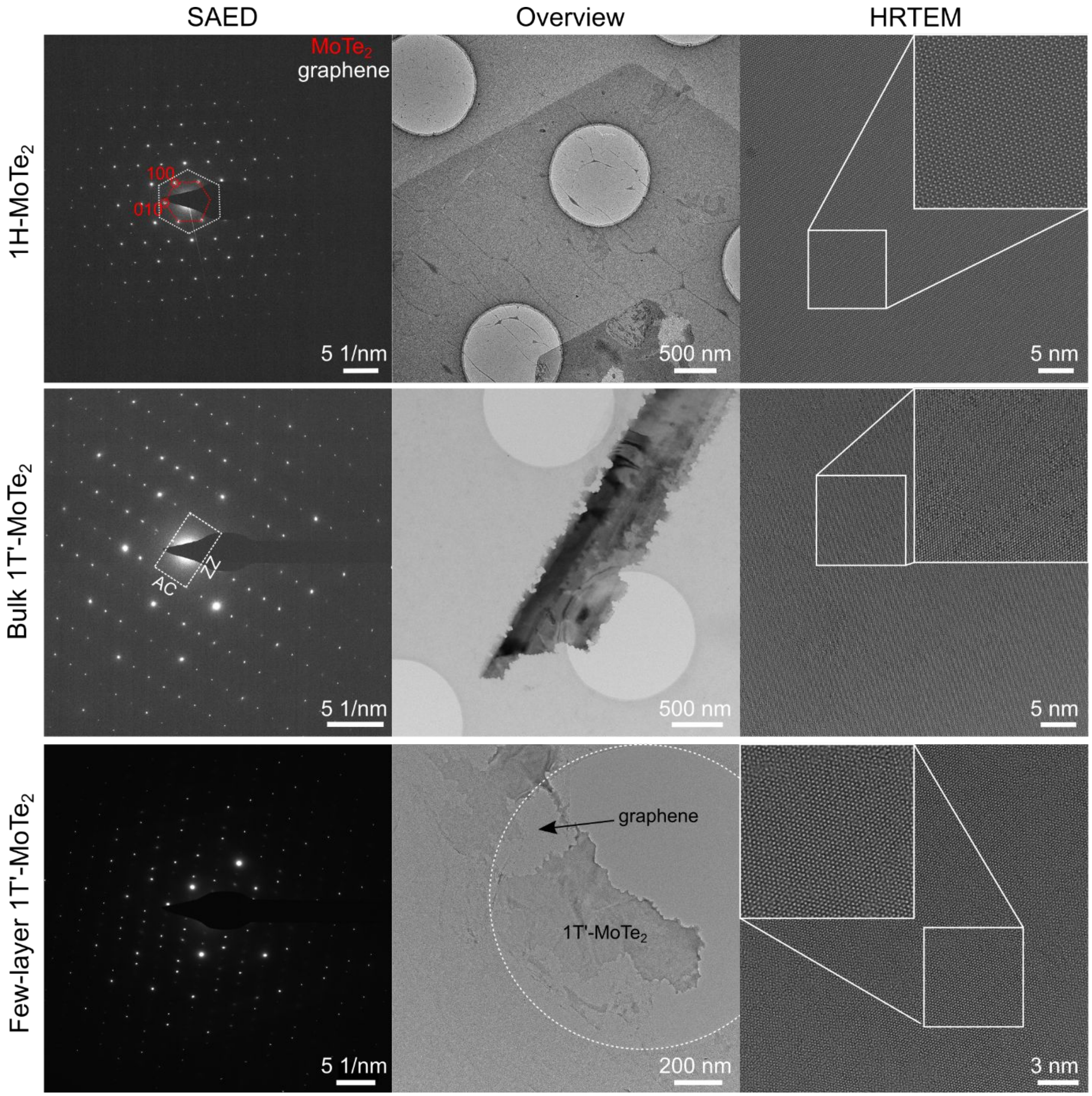

Figure S11. 80 kV SAED patterns, TEM overview images and HRTEM images of monolayer G/1H-$MoTe_2$, bulk 1T'-$MoTe_2$ and few-layer G/1T'-$MoTe_2$. Note: Whereas in the case of monolayer 1H-$MoTe_2$ sharp edges were present in the transferred flakes (see overview image first row), which could also be studied in detail, a clear degradation of the edges is already visible in the case of the transferred bulk 1T'-$MoTe_2$ (see overview image second row). Few-layer as well as monolayer 1T' layers could only be transferred and analyzed in the TEM if they were encapsulated in graphene. Therefore, we conclude that the 1T' flakes are extremely sensitive to our TEM sample preparation.

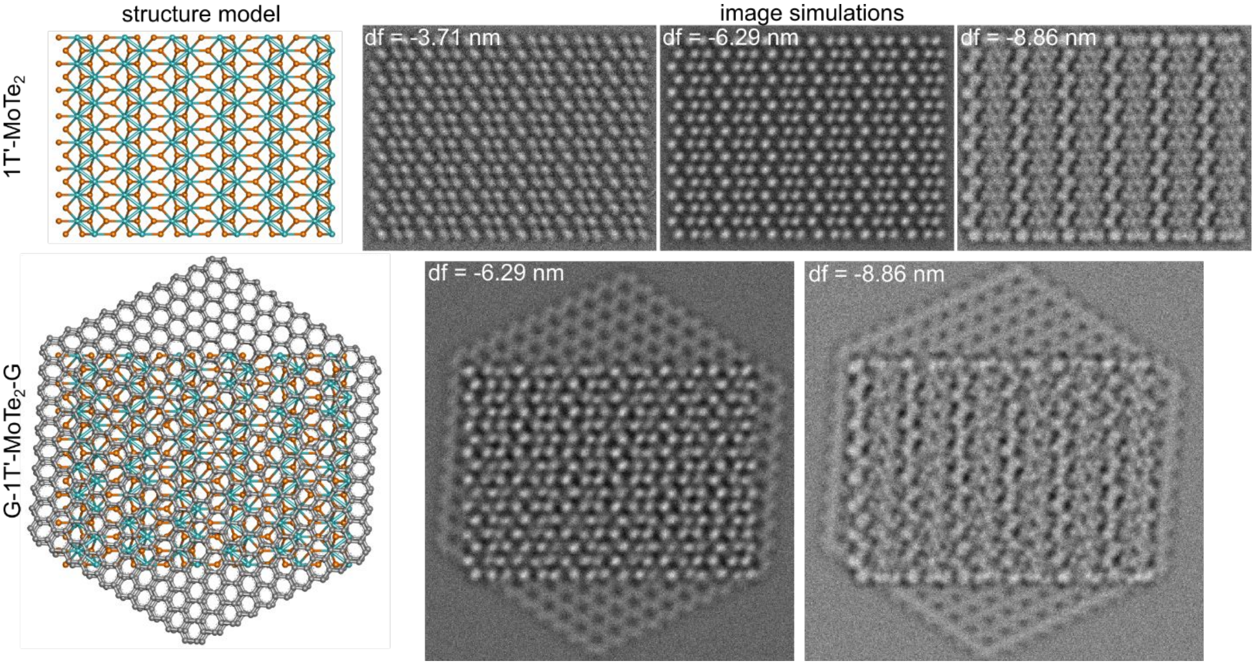

Figure S12. Structure models of single-layer 1T’-$MoTe_2$ (first row) and graphene-encapsulated 1T’-$MoTe_2$ (second row). Besides are image simulations of the structure models for different defoci (df). From the image simulations, it is clear that slight defocus changes and graphene-encapsulation strongly influence image contrast. Therefore, a quantitative fit of the simulations to the experimental images is rather difficult. All image simulations were performed using the open-source abTEM Python Application Programming Interface (API) [5].

9. Transition temperature and chemical stabilization.

To date, the 1H to 1T’ phase transition phenomenon induced by thermal annealing has been investigated by few works. The transition temperature was calculated by Yuan et al. to be 927 ^{o}C [6] using only vibrational contribution to the Helmholtz Free energy. Rehn et al. performed theoretical calculations with vibrational and electronic contributions that resulted in a transition temperature of 417 ^{o}C [7], while experimentally it was measured by Ryu et al. to be 1075 ^{o}C for hBN encapsulated monolayer $MoTe_2$ [8]. The discrepancy between theoretical and experimental results can be attributed to the fact that the ground state energy difference between the two phases in the experiment is higher than the value (i.e., 30-60 meV/f.u.) predicted theoretically. A possible explanation for this could be that the substrate does not allow free cell relaxation of $MoTe_2$ during the phase transformation which, instead, occurs in the constant cell regime [9]. Ryu et al. also experimentally observed a lack of reversibility in the phase transition upon subsequent annealing [8]. To better understand the sole effect of temperature on the phase transition phenomenon, additional experimental and theoretical studies are hence demanded. Considering the complexity of the 1H to 1T’ phase transition that we observe during the CVD growth, there is no clear way to quantitatively analyze the energy balance. However, qualitatively we would expect SiO_2 to slightly increase the energy barrier between the phases and thus stabilize the 1H phase, as it was calculated by Naylor et al [10]. This is in contrast with what we observe. Yet, one should consider that in our experiment we adopt a complex chemistry in the precursors. In literature, upon chemical complication of the precursor from Mo to MoO_3 + NaCl, phase selectivity can be achieved by varying growth parameters. Fraser et al. demonstrated a difference between Mo and MoO_3 precursors transformation into $MoTe_2$ [11].

The Mo film directly transforms into 2H-$MoTe_2$ during the growth, while MoO_3 initially transforms into 1T' and upon $FeTe_2$ flux increment it transforms into 2H phase [11]. A similar difference was observed by Zhang et al. [12] between MoO_3 and $MoO_{2-2.5}$ precursors. The latter could be transformed into 2H phase upon changes in growth time and temperature, while MoO_3 yielded only 1T' phase at the same growth parameters. Recent work of Guo et al. demonstrated phase selectivity in mono- and bilayer $MoTe_2$ grown by CVD [13]. They report that a temperature of 780 ^oC is required to stabilize the 2H phase as well as a ratio between MoO_3 and NaCl powders higher than 8:1. On the other hand, they report that the 1T' phase is dominating at 740 ^oC and 4:1 powders ratio, thus observing 1T' phase stabilization at a lower temperature than those reported for exfoliated flakes [8]. In our work we report coexistence of 1H and 1T' phases at a temperature of 730 ^oC, and in general we observe dominating 1T' phase at those temperatures [14]. Such results could be explained by the presence of O and Na adatoms which have been theoretically predicted to stabilize the 1T' phase [15]. Indeed, in our work, we use AHM as the precursor, which exhibits multiple transformations into different MoO_x upon annealing [16]. Kim et al. reported that AHM preferentially decomposes into Na_2MoO_4 during MoS_2 CVD growth, resulting in a higher number of Na and O atoms per Mo atom [17] than those potentially present in growth chemistries such as those reported in [11–13]. Wienold et al. declare that: "Under a hydrogen containing atmosphere, a peculiar decomposition pathway is observed: an intermediate MoO_3 with an unusual texture is formed prior to the reduction to MoO_2 and the consecutive formation of orthorhombic Mo_4O_{11}" [16]. By carefully considering what discussed until now, a plausible scenario in our experiments could be the following. We expect the formation of MoOx with lower oxygen number to lead to localized 1H phase stabilization, while transformation of AHM into Na_2MoO_4 favors 1T' phase stabilization. We expect that a proper decomposition control of AHM or O and Na atoms binding agent addition to the precursor may lead to the stabilization of the monolayer 1H phase during the CVD growth.

10. Theory and simulations.

To estimate strain contribution on the phase transition triggered by the heterocontact formation we performed DFT simulations for strain applied along both AC and ZZ directions (see Figure S13 (a, b)). We kept desirable axis fixed at certain values while allowing cell relaxation in the orthogonal direction. For the heterocontact strain values, obtained from the direct contact simulations, we do not see significant improvement in the energy difference between 1H and 1T' ground states. Note, that keeping constant cell upon phase transformation leads to the 1H-1T' energy difference increase and results in higher strain required for phase transition. To get an angular dependence of the applied strain we have used the rotational 2D transformation of the Cauchy stress tensor to obtain the orientation-dependent Young's modulus which then was applied for $MoTe_2$ and Janus MoSTe in the work [18]:

$$Y(\alpha) = \frac{C_{11}C_{22} - C_{12}^2}{C_{22}\sin^4(\alpha) + \left(\frac{C_{11}C_{22} - C_{12}^2}{C_{66}} - 2C_{12}\right)\cos^2(\alpha)\sin^2(\alpha) + C_{11}\cos^4(\alpha)} \quad (4)$$

Where α is the angle between ZZ direction and desirable direction **n** (see Figure S13 (c)), $Y(\alpha)$ is the longitudinal orientation-dependent Young's modulus, C_{ij} are elastic constants extracted by applying different strains as described in [19] and summarized in the Table S3 for both phases. For 2H-$MoTe_2$ thermal expansion coefficient is $8.5 \cdot 10^{-6}$ K^{-1} [20], which is equal to approximately 0.66 % biaxial strain at 730 ˚C and can give a small contribution to the AC direction PT while increasing the amount of compressive strain

required to the PT in ZZ direction [21]. In Figure S13, (d) plotted angle dependent 1H-1T' energy difference upon deformations in different directions. Considering both thermal expansion and heterocontact strain contributions we would expect the following scenarios:

a) Contact strain is not sufficient to stabilize 1T' phase at 1000 K and we won't see the 1H to 1T' transformation, but could be opposite effect if the barrier is low but 1H stabilized, the triggering 1T' crystal could be transformed to 1H;
b) Contact strain is partially sufficient for specific angles (i.e. when ZZ directions of incident and transformed crystals are collinear), that should result in a maximum in Figure 3, (f) of the manuscript and partially 1T' to 1H transformation as in a);
c) Contact strain is sufficient and results in 1T' stabilization for all angles.

We do not see a) and b) and our experimental data suggests that c) is a more probable scenario. However, reported theoretical results do not allow us to conclude that the strain is a main mechanism for 1T' stabilization in our case.

Table S3. Elastic constants

C_{ij} (eV/f.u.)	1H	1T'
C_{11}	63	41
C_{12}	17	24
C_{22}	63	69
C_{44}	23	24

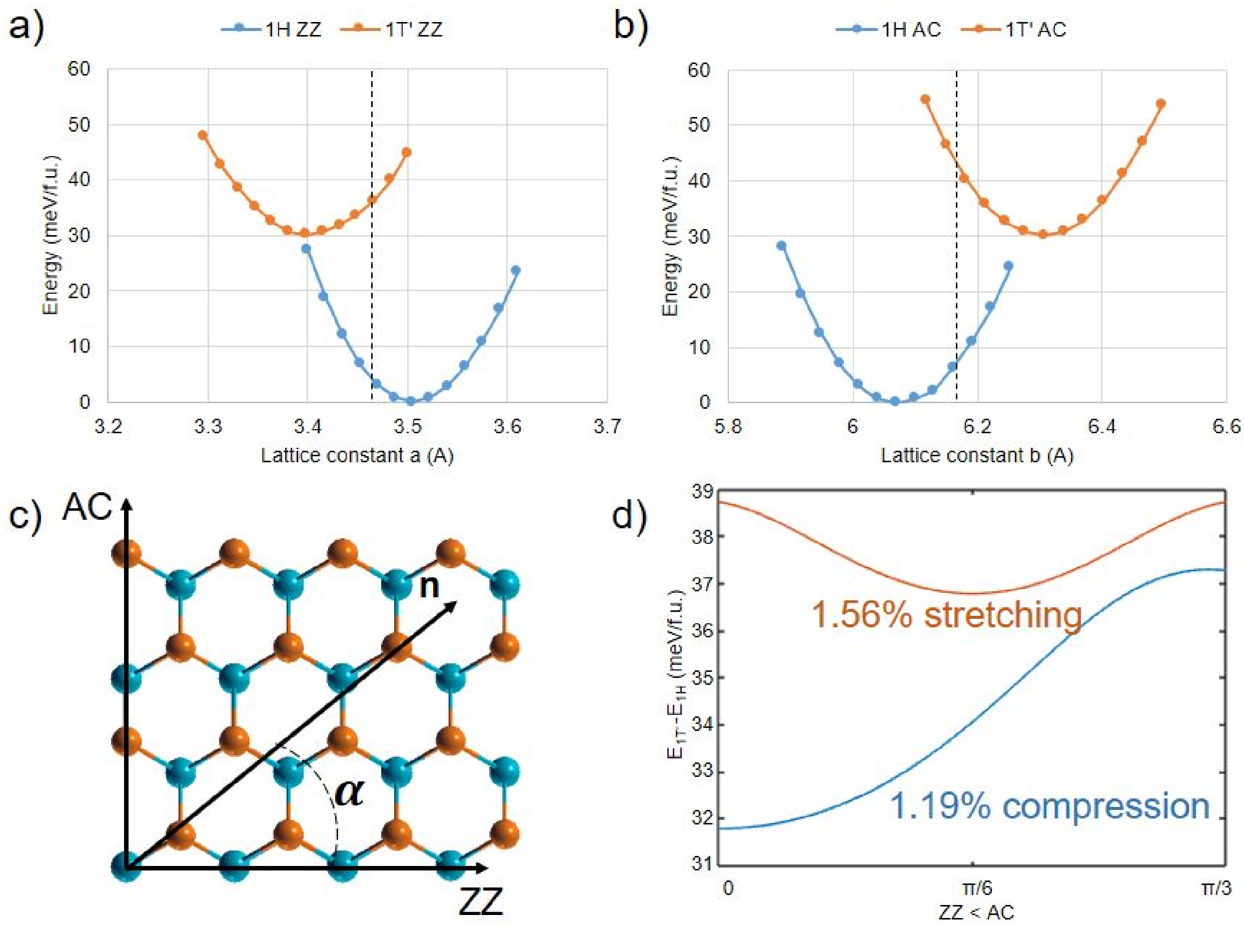

Figure S13. 1H and 1T' ground state energy difference for strain applied in ZZ (a) and AC (b) directions. c) Ball-and-stick model (blue/yellow balls correspond to Mo/Te atoms) with **n** vector showing the strain direction. d) Calculated results for the 1.19% compression and 1.56% stretching corresponding to the HC values obtained from the simulations. AC stretching, which corresponds to $\pi/6$ angle, and ZZ compression, which corresponds to 0 angle, result in the minimum energy difference between the phases. Upon annealing we would expect AC stretching to give slightly higher contribution in the energy difference due to the thermal expansion. Note, that constant cell condition, would give approximately 2 times higher energy difference required for phase transition.

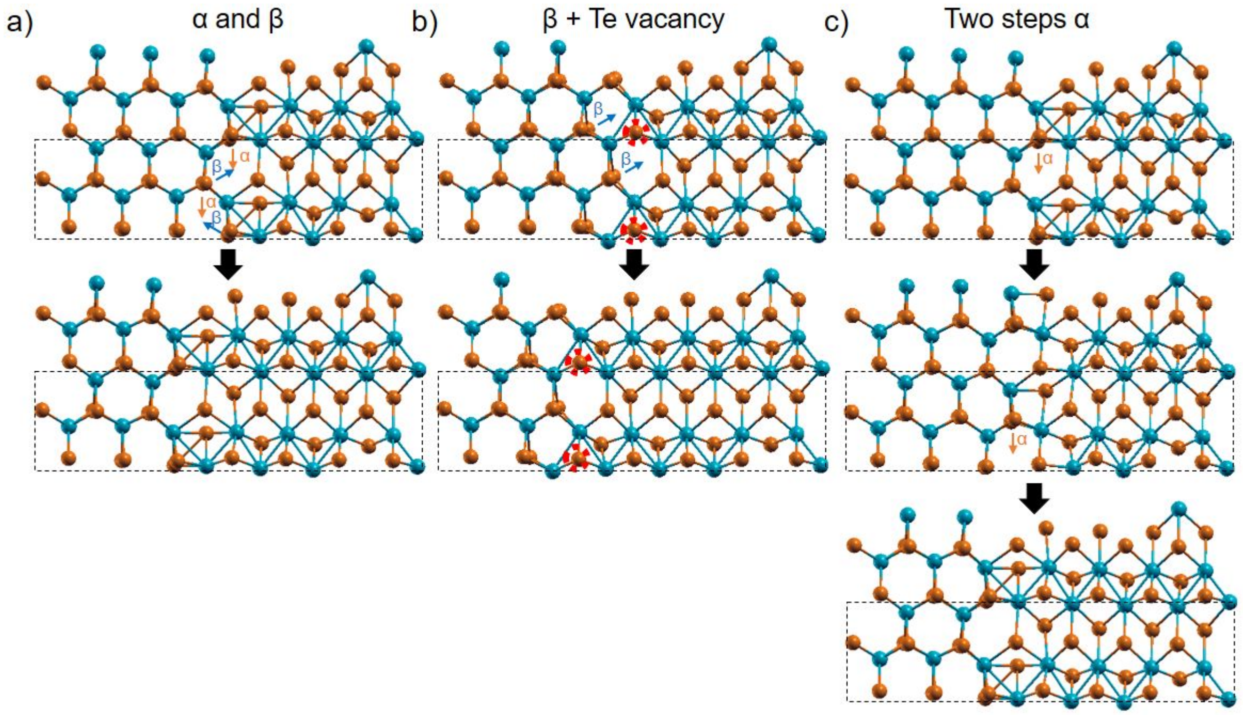

Figure S14. Propagation paths in ZZ direction from Figure 6 of the main text. Arrows indicate lower plane Te atoms displacement direction from initial to final position. Initial and final images of pristine α- and β-paths (a), β-path in the presence of Te vacancy (indicated by red dashed circle) shown in (b), another possible α-path, which can be realized via intermediate state (c). Note, that in case of (b) Te vacancies also diffuse along with 1H-1T' phase transition front that can result in accumulative effect. Unit cells indicated by black dashed lines. The total number of atoms is 41 (40 in case of Te vacancy).

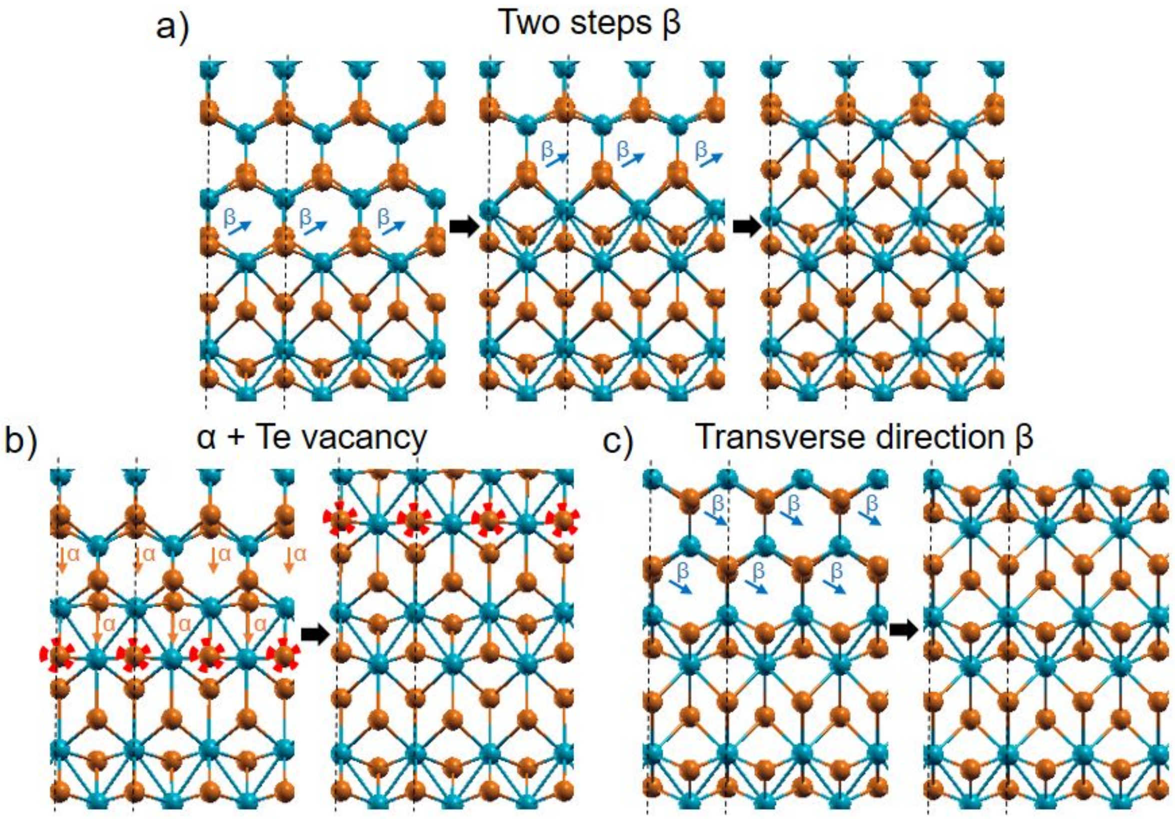

Figure S15. Propagation paths in AC direction from Figure 6 of the main text. Arrows indicate lower plane Te atoms displacement direction from initial to final position. Initial and final states of pristine β-paths, which goes via intermediate state (a), α-path activated by Te vacancy (indicated by red dashed circle) shown in (b), another possible β-path (c), which can be seen as propagation in transverse direction of (a) [22]. Similar to Figure S14, in (b) case, Te vacancies also diffuse along with 1H-1T' phase transition front. Unit cells indicated by black dashed lines. The total number of atoms is 42 (41 in case of Te vacancy).

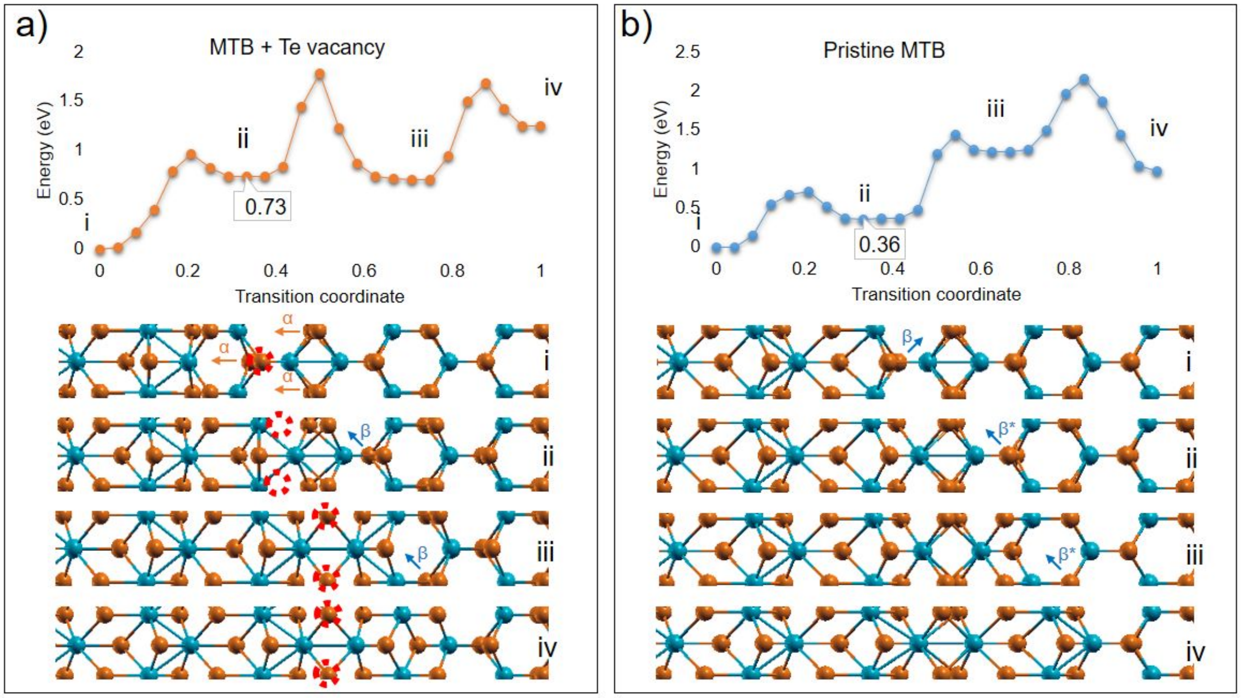

Figure S16. DFT kinetics simulations for the PT in AC direction in presence of MTB defects. In both Te-deficient (a) and pristine (b) cases the PT cannot overcome the MTB and proceed further due to the high energy difference between 1H and formed 1T' phases. β* corresponds to the upper plane Te atoms displacement instead of lower. The total number of atoms is 41 (40 in case of Te vacancy).

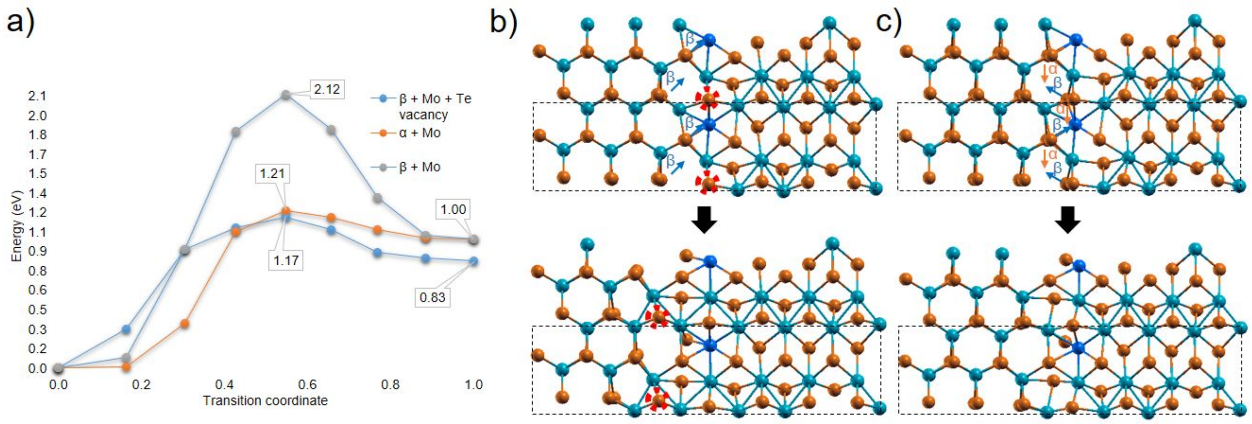

Figure S17. a) DFT simulations for the collinear contact in case of ZZ direction with excess Mo. Simulations performed for the same paths as in Figure S14 with additional Mo atom (intense blue color) in interstitial position. Te-deficient case shown in (b) while both possible pristine α- and β-paths in (c). In all cases 1T' phase is unstable. Unit cells indicated by black dashed lines. The total number of atoms is 42 (41 in case of Te vacancy).

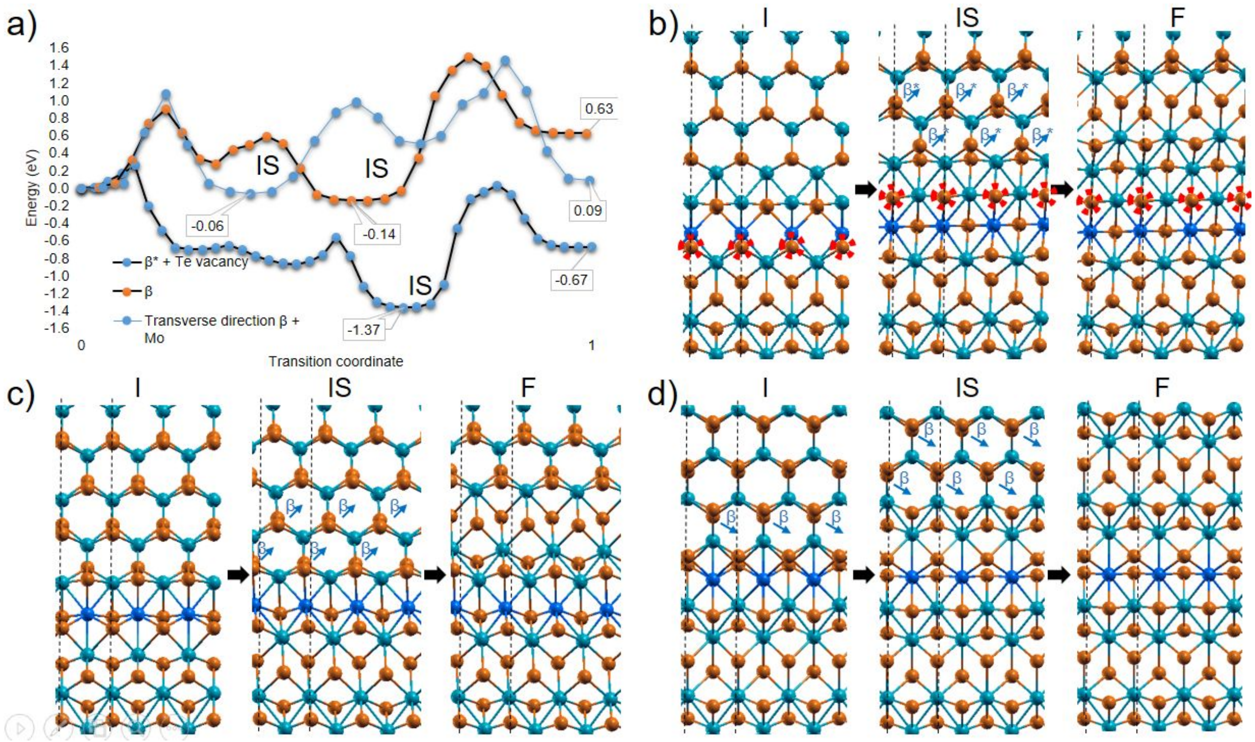

Figure S18. a) DFT simulations for the collinear contact in case of AC direction with excess Mo. Simulations performed for the same paths found in Figure S15 with additional Mo atom in interstitial position. First, we found initial lower energy state and then proceed with different Te atoms displacement in different positions to find possible transformation routes. In all cases, an intermediate state (IS) was found to be lowest energy state. Moreover, in these IS, Mo excess may affect the system similar as double Te vacancies and result in double Mo chains formation similar to previously reported M_4X_6 in other TMDS [23]. We found it in both Te-deficient β* (b) and pristine β (c) cases. β* corresponds to the upper plane Te atoms displacement instead of lower. In case of propagation in transverse direction β (d), no double Mo chains present and instead MTB configuration formed different from case presented in Figure S16 with following cell transformation being 0.15 eV higher than IS energy. Unit cells indicated by black dashed lines. The total number of atoms is 43 (42 in case of Te vacancy)

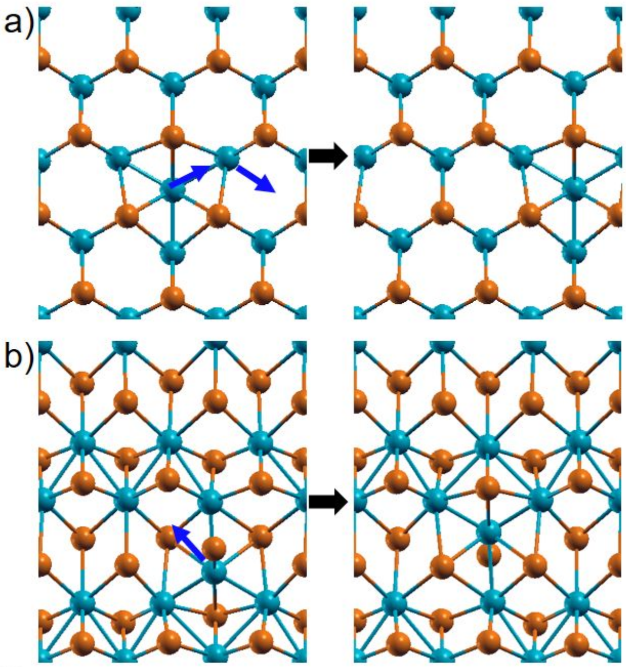

Figure S19. Schematic of a Mo excess diffusion in case of 1H (a) and 1T’ (b) phases. Mo atoms migration direction shown by blue arrows and black arrows connect initial and final configurations. The diffusion mechanism is substitutional in the 1H phase [24], while in the 1T’ it is interstitial. The total number of atoms is 37.

11. **Devices based on 2H-1T’ planar heterostructures**

Phase-patterned $MoTe_2$ could be of interesting to develop FETs with semiconductive 1H-phase channels and 1T’-phase metallic contacts. Such semiconductor-semimetal junctions possess smaller contact resistance and lower Schottky barrier values compared to FETs with fabricated metallic contacts. In the case of monolayer 1H-$MoTe_2$ in contact with different metals, the Schottky barrier for holes was calculated to be 0.5-0.9 eV [25], while experimentally it was measured to be 0.12-0.47 at various temperatures (0.03 to 0.27 eV for electrons) [26]. Planar heterophase 1H-1T’ FETs have not yet been demonstrated, however, in the layered limit Schottky barriers as low as 6 to 30 meV have been reported, as can be seen in Table S4. There, the experimental thickness, transport type, mobility, On/Off current ratio, contact resistance and Schottky barrier for H-T’ junction based FETs are summarized. Noteworthy, the reported 2H-1T’ Schottky barriers are 1-2 orders of magnitude lower than the measured values for the reference metallic contacts [12,27,28].

Table S4. Electrical properties of FETs based on the 2H-1T’ lateral junctions

Thickness	Transport type	Mobility, cm^2/Vs	On/Off current ratio	Contact resistance, kΩ µm	Schottky barrier, meV

>3 layers [27]	P	15	10^5-10^7 (300-77K)	14	22
3.5 nm [28]	P	7.4	$3.5\cdot10^3$ (77K)	-	30
4.2 nm, [29]	P	30	10^4 (300K)	-	-
2 layers [8]	Ambipolar	7.86 (h)	10^6 (300K)	0.372	-
3.2 nm [30]	Ambipolar	16.11 (e)<br>3.9 (h)	10^7 (300K)	1.13	-
2-20nm [12]	P	30-130	10^3-10^5 (300K)	-	20
>10 nm [31]	P	45	$1.8\cdot10^4$ (300K)	1.6	6.15
5 nm [32]	P	23	10^4 (300K)	1.1	-

1T’-2H planar heterojunctions are also an enticing platform to develop photodetectors. Wang et al. reported that photodetectors based on 1T’-2H heterostructures exhibit faster photoresponse and larger photocurrents than those measured for devices realized with the pristine 2H phase [33]. The measurements were performed under 520 nm light illumination and the calculated photoresponsivity was $2.8\cdot10^{-4}$ A/W. The rise and fall times of this photodetector are 384 and 473 µs, respectively. Xu et al, discussed the adoption of 2H-1T’ heterojunctions in NIR flexible and transparent optoelectronics [32]. They demonstrated a NIR photodetector operating under 1064 nm light illumination with 1.02 A/W photoresponsivity.

To further corroborate that the lateral 1H-1T’ heterostructure reported in this work is promising for the realization of low contact resistance devices, Kelvin probe force microscopy (KPFM) measurements were performed. KPFM is widely used to estimate the work function difference between physically contacting materials. In case of layered $MoTe_2$, the reported surface potential difference between 2H and 1T’ ranges from 25 [29] to 100 [34] mV. We performed KPFM measurements on our SiO_2/$MoTe_2$/hBN samples and found a surface potential difference between the 1H and 1T’ phases of about 118 mV (see Figure S20). We should note that this measurement is not a direct work function difference measurement because we measure “floating” samples in between dielectric SiO_2 and hBN and should be treated only as an indirect evidence of the low surface potential difference.

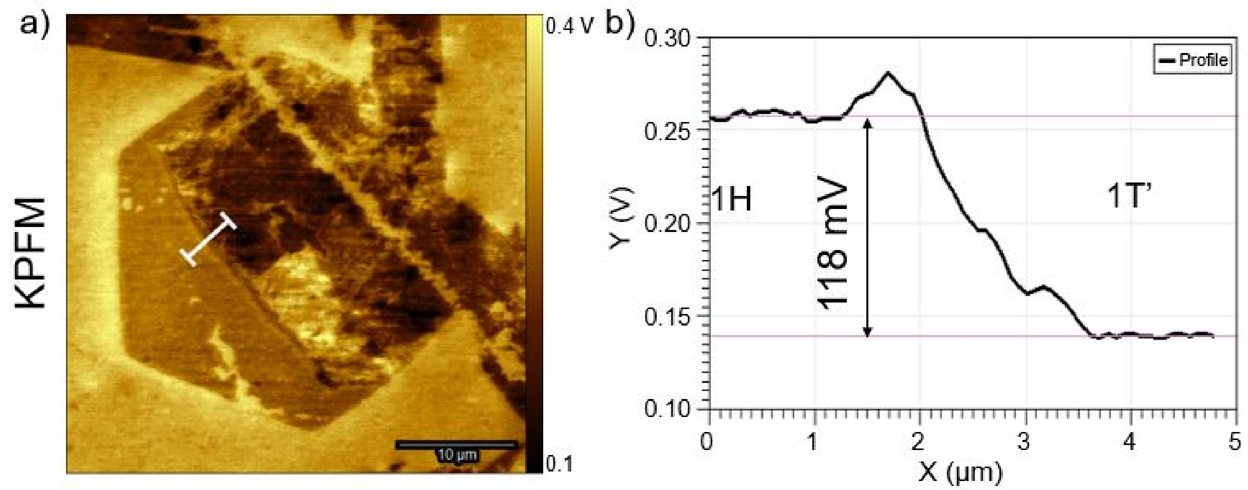

Figure S20. a) KPFM measurement of the sample described in Figure 2 of the main text. b) Surface potential profile extracted along 1H-1T' heterointerface in (a).

References

(1) Hofmann, S. *Auger- and X-Ray Photoelectron Spectroscopy in Materials Science: A User-Oriented Guide*; Springer Science & Business Media, 2012.

(2) Yeh, J. J.; Lindau, I. Atomic Subshell Photoionization Cross Sections and Asymmetry Parameters: 1 ⩽ Z ⩽ 103. *At. Data Nucl. Data Tables* **1985**, *32* (1), 1–155. https://doi.org/10.1016/0092-640X(85)90016-6.

(3) Powell, C. J.; Jablonski, A. The NIST Electron Effective-Attenuation-Length Database. *NIST* **2002**.

(4) Nguyen-Truong, H. T.; Da, B.; Yang, L.; Ding, Z.; Yoshikawa, H.; Tanuma, S. Low-Energy Electron Inelastic Mean Free Path for Monolayer Graphene. *Appl. Phys. Lett.* **2020**, *117* (3), 033103. https://doi.org/10.1063/5.0016284.

(5) Madsen, J.; Susi, T. The AbTEM Code: Transmission Electron Microscopy from First Principle. 2021. https://doi.org/10.12688/openreseurope.13015.2.

(6) Yuan, J.; Chen, Y.; Xie, Y.; Zhang, X.; Rao, D.; Guo, Y.; Yan, X.; Feng, Y. P.; Cai, Y. Squeezed Metallic Droplet with Tunable Kubo Gap and Charge Injection in Transition Metal Dichalcogenides. *Proc. Natl. Acad. Sci.* **2020**, *117* (12), 6362–6369. https://doi.org/10.1073/pnas.1920036117.

(7) Rehn, D. A.; Li, Y.; Pop, E.; Reed, E. J. Theoretical Potential for Low Energy Consumption Phase Change Memory Utilizing Electrostatically-Induced Structural Phase Transitions in 2D Materials. *Npj Comput. Mater.* **2018**, *4* (1), 2. https://doi.org/10.1038/s41524-017-0059-2.

(8) Ryu, H.; Lee, Y.; Kim, H.; Kang, S.; Kang, Y.; Kim, K.; Kim, J.; Janicek, B. E.; Watanabe, K.; Taniguchi, T.; Huang, P. Y.; Cheong, H.; Jung, I.; Kim, K.; Son, Y.; Lee, G. Anomalous Dimensionality-Driven Phase Transition of $MoTe_2$ in Van Der Waals Heterostructure. *Adv. Funct. Mater.* **2021**, *31* (51), 2107376. https://doi.org/10.1002/adfm.202107376.

(9) Li, Y.; Duerloo, K.-A. N.; Wauson, K.; Reed, E. J. Structural Semiconductor-to-Semimetal Phase Transition in Two-Dimensional Materials Induced by Electrostatic Gating. *Nat. Commun.* **2016**, *7* (1), 10671. https://doi.org/10.1038/ncomms10671.

(10) Naylor, C. H.; Parkin, W. M.; Ping, J.; Gao, Z.; Zhou, Y. R.; Kim, Y.; Streller, F.; Carpick, R. W.; Rappe, A. M.; Drndić, M.; Kikkawa, J. M.; Johnson, A. T. C. Monolayer Single-Crystal 1T'-MoTe2 Grown by

Chemical Vapor Deposition Exhibits Weak Antilocalization Effect. *Nano Lett.* **2016**, *16* (7), 4297–4304. https://doi.org/10.1021/acs.nanolett.6b01342.

(11) Fraser, J. P.; Masaityte, L.; Zhang, J.; Laing, S.; Moreno-López, J. C.; McKenzie, A.; McGlynn, J.; Panchal, V.; Graham, D.; Kazakova, O.; Pichler, T.; MacLaren, D. A.; Moran, D. A. J.; Ganin, A. Y. *A Universal Platform for Selective Phase Growth and Precise-Layer Control in MoTe2*; preprint; Chemistry, 2020. https://doi.org/10.26434/chemrxiv.11882547.v1.

(12) Zhang, Q.; Wang, X.-F.; Shen, S.-H.; Lu, Q.; Liu, X.; Li, H.; Zheng, J.; Yu, C.-P.; Zhong, X.; Gu, L.; Ren, T.-L.; Jiao, L. Simultaneous Synthesis and Integration of Two-Dimensional Electronic Components. *Nat. Electron.* **2019**, *2* (4), 164–170. https://doi.org/10.1038/s41928-019-0233-2.

(13) Guo, Z.; Wang, L.; Han, M.; Zhao, E.; Zhu, L.; Guo, W.; Tan, J.; Liu, B.; Chen, X.-Q.; Lin, J. One-Step Growth of Bilayer 2H–1T′ MoTe2 van Der Waals Heterostructures with Interlayer-Coupled Resonant Phonon Vibration. *ACS Nano* **2022**, *16* (7), 11268–11277. https://doi.org/10.1021/acsnano.2c04664.

(14) Pace, S.; Martini, L.; Convertino, D.; Keum, D. H.; Forti, S.; Pezzini, S.; Fabbri, F.; Mišeikis, V.; Coletti, C. Synthesis of Large-Scale Monolayer 1T′-MoTe2 and Its Stabilization via Scalable HBN Encapsulation. *ACS Nano* **2021**, *15* (3), 4213–4225. https://doi.org/10.1021/acsnano.0c05936.

(15) Zhou, Y.; Reed, E. J. Structural Phase Stability Control of Monolayer $MoTe_2$ with Adsorbed Atoms and Molecules. *J. Phys. Chem. C* **2015**, *119* (37), 21674–21680. https://doi.org/10.1021/acs.jpcc.5b05770.

(16) Wienold, J.; Jentoft, R. E.; Ressler, T. Structural Investigation of the Thermal Decomposition of Ammonium Heptamolybdate by in Situ XAFS and XRD. *Eur. J. Inorg. Chem.* **2003**, *2003* (6), 1058–1071. https://doi.org/10.1002/ejic.200390138.

(17) Kim, H.; Han, G. H.; Yun, S. J.; Zhao, J.; Keum, D. H.; Jeong, H. Y.; Ly, T. H.; Jin, Y.; Park, J.-H.; Moon, B. H.; Kim, S.-W.; Lee, Y. H. Role of Alkali Metal Promoter in Enhancing Lateral Growth of Monolayer Transition Metal Dichalcogenides. *Nanotechnology* **2017**, *28* (36), 36LT01. https://doi.org/10.1088/1361-6528/aa7e5e.

(18) Yagmurcukardes, M.; Sevik, C.; Peeters, F. M. Electronic, Vibrational, Elastic, and Piezoelectric Properties of Monolayer Janus MoSTe Phases: A First-Principles Study. *Phys. Rev. B* **2019**, *100* (4), 045415. https://doi.org/10.1103/PhysRevB.100.045415.

(19) Cadelano, E.; Palla, P. L.; Giordano, S.; Colombo, L. Elastic Properties of Hydrogenated Graphene. *Phys. Rev. B* **2010**, *82* (23), 235414. https://doi.org/10.1103/PhysRevB.82.235414.

(20) Wang, Z.-Y.; Zhou, Y.-L.; Wang, X.-Q.; Wang, F.; Sun, Q.; Guo, Z.-X.; Jia, Y. Effects of In-Plane Stiffness and Charge Transfer on Thermal Expansion of Monolayer Transition Metal Dichalcogenide*. *Chin. Phys. B* **2015**, *24* (2), 026501. https://doi.org/10.1088/1674-1056/24/2/026501.

(21) Duerloo, K.-A. N.; Li, Y.; Reed, E. J. Structural Phase Transitions in Two-Dimensional Mo- and W-Dichalcogenide Monolayers. *Nat. Commun.* **2014**, *5* (1), 4214. https://doi.org/10.1038/ncomms5214.

(22) Ghasemi, A.; Gao, W. Atomistic Mechanism of Stress Modulated Phase Transition in Monolayer MoTe 2. *Extreme Mech. Lett.* **2020**, *40*, 100946. https://doi.org/10.1016/j.eml.2020.100946.

(23) Hong, J.; Chen, X.; Li, P.; Koshino, M.; Li, S.; Xu, H.; Hu, Z.; Ding, F.; Suenaga, K. Multiple 2D Phase Transformations in Monolayer Transition Metal Chalcogenides. *Adv. Mater.* **2022**, *34* (19), 2200643. https://doi.org/10.1002/adma.202200643.

(24) Coelho, P. M.; Komsa, H.-P.; Coy Diaz, H.; Ma, Y.; Krasheninnikov, A. V.; Batzill, M. Post-Synthesis Modifications of Two-Dimensional MoSe2 or MoTe2 by Incorporation of Excess Metal Atoms into the Crystal Structure. *ACS Nano* **2018**, *12* (4), 3975–3984. https://doi.org/10.1021/acsnano.8b01580.

(25) Guo, Y.; Liu, D.; Robertson, J. 3D Behavior of Schottky Barriers of 2D Transition-Metal Dichalcogenides. *ACS Appl. Mater. Interfaces* **2015**, *7* (46), 25709–25715. https://doi.org/10.1021/acsami.5b06897.
(26) Kim, C.; Moon, I.; Lee, D.; Choi, M. S.; Ahmed, F.; Nam, S.; Cho, Y.; Shin, H.-J.; Park, S.; Yoo, W. J. Fermi Level Pinning at Electrical Metal Contacts of Monolayer Molybdenum Dichalcogenides. *ACS Nano* **2017**, *11* (2), 1588–1596. https://doi.org/10.1021/acsnano.6b07159.
(27) Sung, J. H.; Heo, H.; Si, S.; Kim, Y. H.; Noh, H. R.; Song, K.; Kim, J.; Lee, C.-S.; Seo, S.-Y.; Kim, D.-H.; Kim, H. K.; Yeom, H. W.; Kim, T.-H.; Choi, S.-Y.; Kim, J. S.; Jo, M.-H. Coplanar Semiconductor–Metal Circuitry Defined on Few-Layer MoTe2 via Polymorphic Heteroepitaxy. *Nat. Nanotechnol.* **2017**, *12* (11), 1064–1070. https://doi.org/10.1038/nnano.2017.161.
(28) Ma, R.; Zhang, H.; Yoo, Y.; Degregorio, Z. P.; Jin, L.; Golani, P.; Ghasemi Azadani, J.; Low, T.; Johns, J. E.; Bendersky, L. A.; Davydov, A. V.; Koester, S. J. MoTe2 Lateral Homojunction Field-Effect Transistors Fabricated Using Flux-Controlled Phase Engineering. *ACS Nano* **2019**, *13* (7), 8035–8046. https://doi.org/10.1021/acsnano.9b02785.
(29) Xu, X.; Chen, S.; Liu, S.; Cheng, X.; Xu, W.; Li, P.; Wan, Y.; Yang, S.; Gong, W.; Yuan, K.; Gao, P.; Ye, Y.; Dai, L. Millimeter-Scale Single-Crystalline Semiconducting $MoTe_2$ via Solid-to-Solid Phase Transformation. *J. Am. Chem. Soc.* **2019**, *141* (5), 2128–2134. https://doi.org/10.1021/jacs.8b12230.
(30) Ryu, H.; Lee, Y.; Jeong, J. H.; Lee, Y.; Cheon, Y.; Watanabe, K.; Taniguchi, T.; Kim, K.; Cheong, H.; Lee, C.-H.; Lee, G.-H. Laser-Induced Phase Transition and Patterning of HBN-Encapsulated Mo Te2. *Small n/a* (n/a), 2205224. https://doi.org/10.1002/smll.202205224.
(31) Xu, X.; Pan, Y.; Liu, S.; Han, B.; Gu, P.; Li, S.; Xu, W.; Peng, Y.; Han, Z.; Chen, J.; Gao, P.; Ye, Y. Seeded 2D Epitaxy of Large-Area Single-Crystal Films of the van Der Waals Semiconductor 2H $MoTe_2$. *Science* **2021**, *372* (6538), 195–200. https://doi.org/10.1126/science.abf5825.
(32) Xu, X.; Liu, S.; Han, B.; Han, Y.; Yuan, K.; Xu, W.; Yao, X.; Li, P.; Yang, S.; Gong, W.; Muller, D. A.; Gao, P.; Ye, Y.; Dai, L. Scaling-up Atomically Thin Coplanar Semiconductor–Metal Circuitry via Phase Engineered Chemical Assembly. *Nano Lett.* **2019**, *19* (10), 6845–6852. https://doi.org/10.1021/acs.nanolett.9b02006.
(33) Wang, Y.; Zhang, M.; Xue, Z.; Chen, X.; Mei, Y.; Chu, P. K.; Tian, Z.; Wu, X.; Di, Z. Atomistic Observation of the Local Phase Transition in $MoTe_2$ for Application in Homojunction Photodetectors. *Small* **2022**, *18* (19), 2200913. https://doi.org/10.1002/smll.202200913.
(34) Yoo, Y.; DeGregorio, Z. P.; Su, Y.; Koester, S. J.; Johns, J. E. In-Plane 2H-1T′ MoTe2 Homojunctions Synthesized by Flux-Controlled Phase Engineering. *Adv. Mater.* **2017**, *29* (16), 1605461. https://doi.org/10.1002/adma.201605461.
